# Large Sex Differences in Chicken Behavior and Brain Gene Expression Coincide with Few Differences in Promoter DNA-Methylation

**DOI:** 10.1371/journal.pone.0096376

**Published:** 2014-04-29

**Authors:** Daniel Nätt, Beatrix Agnvall, Per Jensen

**Affiliations:** 1 IFM Biology, AVIAN Behaviour and Genomics group, Linköping University, Linköping, Sweden; 2 Department of Clinical and Experimental Medicine, Laboratory of Integrative and Behavioral Neuroscience, Linköping University, Linköping, Sweden; University of Nevada School of Medicine, United States of America

## Abstract

While behavioral sex differences have repeatedly been reported across taxa, the underlying epigenetic mechanisms in the brain are mostly lacking. Birds have previously shown to have only limited dosage compensation, leading to high sex bias of Z-chromosome gene expression. In chickens, a male hyper-methylated region (MHM) on the Z-chromosome has been associated with a local type of dosage compensation, but a more detailed characterization of the avian methylome is limiting our interpretations. Here we report an analysis of genome wide sex differences in promoter DNA-methylation and gene expression in the brain of three weeks old chickens, and associated sex differences in behavior of Red Junglefowl (ancestor of domestic chickens). Combining DNA-methylation tiling arrays with gene expression microarrays we show that a specific locus of the MHM region, together with the promoter for the *zinc finger RNA binding protein* (*ZFR*) gene on chromosome 1, is strongly associated with sex dimorphism in gene expression. Except for this, we found few differences in promoter DNA-methylation, even though hundreds of genes were robustly differentially expressed across distantly related breeds. Several of the differentially expressed genes are known to affect behavior, and as suggested from their functional annotation, we found that female Red Junglefowl are more explorative and fearful in a range of tests performed throughout their lives. This paper identifies new sites and, with increased resolution, confirms known sites where DNA-methylation seems to affect sexually dimorphic gene expression, but the general lack of this association is noticeable and strengthens the view that birds do not have dosage compensation.

## Background

A wide range of strategies for reproduction and social behavior exists among animals. Depending on whether the species lives solitary or in groups, in matriarchates or patriarchates, and in polyandry, polygyny or polygamy, the roles of the two sexes may differ dramatically [1] [2]. This may lead to the evolution of distinctly different behavioral roles of the sexes, shaping behavioral differences of various kinds [1]. Although there are many accounts of such sex specific behavioral roles, very little is known about the genetic mechanisms behind them. As the genomes of males and females of the same species are highly similar (except for the sex chromosomes), we would expect gene expression and epigenetic factors to be important in shaping the phenotypic differences.

Sex specific gene expression has been observed in many taxa such as insects, nematodes, birds and mammals [3]. For instance, approximately 80% of the gene expression which differs between *Drosophila simulans* and *melanogaster* is attributable to sexually dimorphic genes [4]. While few cases have directly linked gene expression with unique behavioral traits, at least in rodents, plenty of genetic and epigenetic differences have been identified in brain regions critical for behavioral control (reviewed in [5]). In mice, sexual dimorphisms in brain gene expression are present even before embryonic hormonal secretion, which suggests functional differences in the brain that are independent of embryonic hormones [6]. However, there is still limited knowledge of the relationship between sex differences in gene expression and epigenetic factors on one hand and the corresponding behavioral differences on the other.

Studies on sex specific epigenetic mechanism often address the concept of dosage compensation. Organisms with heteromorphic sex chromosomes, such as mammals (female:XX, male: XY) and birds (female:ZW, male:ZZ), face a genetic problem since the heterogametic sex (males in mammals and females in birds) only get half the dosage of the dominant sex chromosome, potentially leading to lower expression from genes on this chromosome. In mammals, this loss of dosage is compensated by a general silencing of one of the female X-chromosomes [7]. The *Xist* gene, which transcribes a long non-coding RNA and is expressed only from the silenced chromosome [8], seems to initiate this by mediating histone modifications and promoter methylation of cytosine residues all over the affected chromosome [9] [10]. Unlike mammals, birds seem to lack global mechanisms for dosage compensation leading to highly biased expression of genes on the Z-chromosome (reviewed in [11]). Surprisingly, even though the Z-chromosome contains about 10% of all known genes in the chicken genome, this apparently does not lead to any functional failure of biological systems. Recently it has been suggested that birds partially use mammalian-type dosage compensation to circumvent some of the problems [12] [13]. In this context, a so called male hypermethylated region (MHM) on the Z-chromosome has been identified [14], and just like *Xist* it transcribes a large noncoding RNA that is thought to mediate DNA-methylation locally around itself. Interestingly, it has been suggested that the MHM region may cause down-regulation of the closely linked *DMRT1* gene, which previously was shown to be important for sex determination in species of many taxa including birds [15], [16].

The overall aim of this experiment was to identify epigenetic targets possibly involved in regulating sexually dimorphic behaviors. Specifically, we studied sex differences in gene expression and DNA-methylation on a genome wide scale in the brains of three weeks old chickens and characterized sex differences in behavior. By sampling brains early in adolescence, just prior to when most sexually dimorphic behaviors emerge in the chicken, we hoped to identify key regulators that initiate the sexual differentiation of the brain during this period. By focusing the genetic analysis on thalamus/hypothalamus we primarily aimed to identify gene targets involved in stress regulation and therefore focused on behaviors relevant to stress and fear. To evaluate the generality of our findings, we used two genetically distinct breeds with unique selection histories over several thousand years; the Red Junglefowl (*Gallus gallus*), ancestor of domestic chickens, and the domesticated White Leghorn (*Gallus gallus domesticus*). Both breeds are highly sexually dimorphic, with an excessively ornamented male of about double the size of the more cryptic female [17]. Both sexes are sexually promiscuous, but sexual coercion by the male is often necessary for copulation to occur [18] [19]. While the domesticated breed still expresses most of its natural behaviors, commercial selection for certain sexually relevant characteristics (egg laying frequency, egg size, body mass), has changed the expression level of sexual characters, leading for example to larger but less colorful sex ornaments.

## Results and Discussion

### Gene expression: Limited dosage compensation

To examine epigenetic differences between the sexes, we extracted DNA and mRNA from the same samples, each comprising a part of the brain enriched for hypothalamus and thalamus. Samples were collected from 12 Red Junglefowl (RJF) of each sex when they were 21 days old, and were pooled into two pools per sex for gene expression microarray and DNA-methylation tiling array analysis. An identical sample set was also collected for domesticated White Leghorn chicks (WL). The total number of animals was therefore 24, pooled and hybridized onto eight arrays, four arrays of each sex and breed.

**Figure 1:**
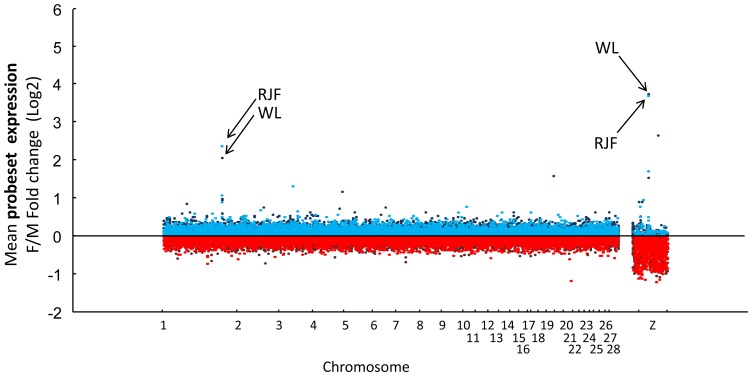
Differential expression of genes in females (F) compared to males (M) in Red Junglefowl (RJF) and White Leghorn (WL) respectively. Each dot represents the mean fold change difference (log 2) of one microarray probeset. Red dots indicate down-regulation in females, and blue dots up-regulation. Clear dots indicate RJF and dark colored dots indicate WL. Arrows indicate the two probesets on chromosome 1 and Z with the strongest fold change signals across breeds within the whole experiment. Genebuild: WASHUC2.

**Figure 2:**
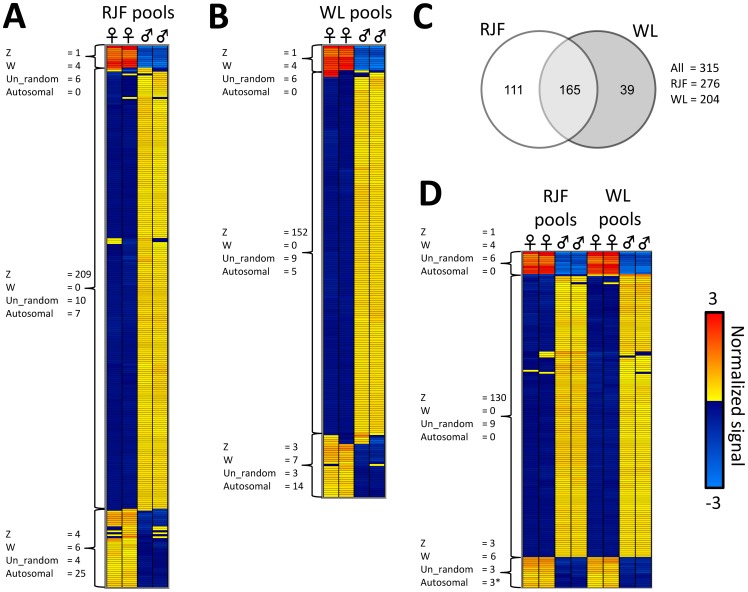
Significant gene expression differences between sexes within and across breeds. Heat map columns represent individual microarrays, while rows represent significantly differentially expressed (DE) probesets adjusted for false discovery rate. Heat maps are organized using hierarchical cluster analysis (average linkage) identifying three major clades both within and across breeds (clades are represented by brackets). **A**) Probesets DE within the Red Junglefowl (RJF pools). **B**) Probesets DE within the White Leghorn (WL pools). **C**) Venn-diagram showing numbers of DE probesets within breeds that are also DE in the other breed. **D**) Probesets DE across breeds. *Note that DE probesets annotated to autosomes are common within breed, but only three probesets remain in the across breed comparison, all annotated to the same gene: *ZFR* on chromosome 1. Each microarray was hybridized with a pool of six birds.

**Figure 3:**
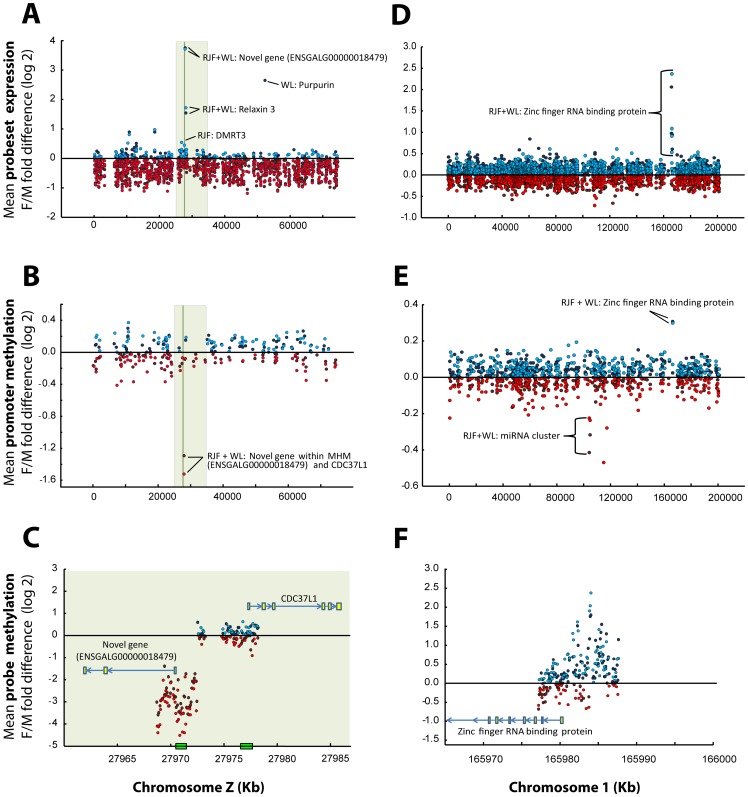
Gene expression and DNA-methylation differences between females (F) and males (M) on chromosome Z and 1 respectively. Dots represent fold change differences (log 2) within three categories: A) and D)  =  Expression microarray probesets; B) and E)  =  DNA-methylation tiling array probes averaged within promoter; C) and F)  =  Individual DNA-methylation tiling array probes. A) shows gene expression differences on the Z chromosome. B) shows promoter methylation differences on the Z-chromosome. C) shows individual probe differences in the promoters of a novel gene close to MHM and *CDC37L1*. D) shows gene expression differences on chromosome 1. E) shows promoter methylation differences on chromosome 1. F) shows individual probe differences in the promoter to *ZFR* on chromosome 1. Red dots signify down-regulation in females, and blue dots up-regulation. Clear dots indicate Red Junglefowl (RJF) and dark colored dots White Leghorn (WL). The clear green shaded area represents the dosage compensated region reported by [12], and the dark green line represent the location of the male hyper methylated region reported by [14]. Genebuild: WASHUC2.

**Figure 4:**
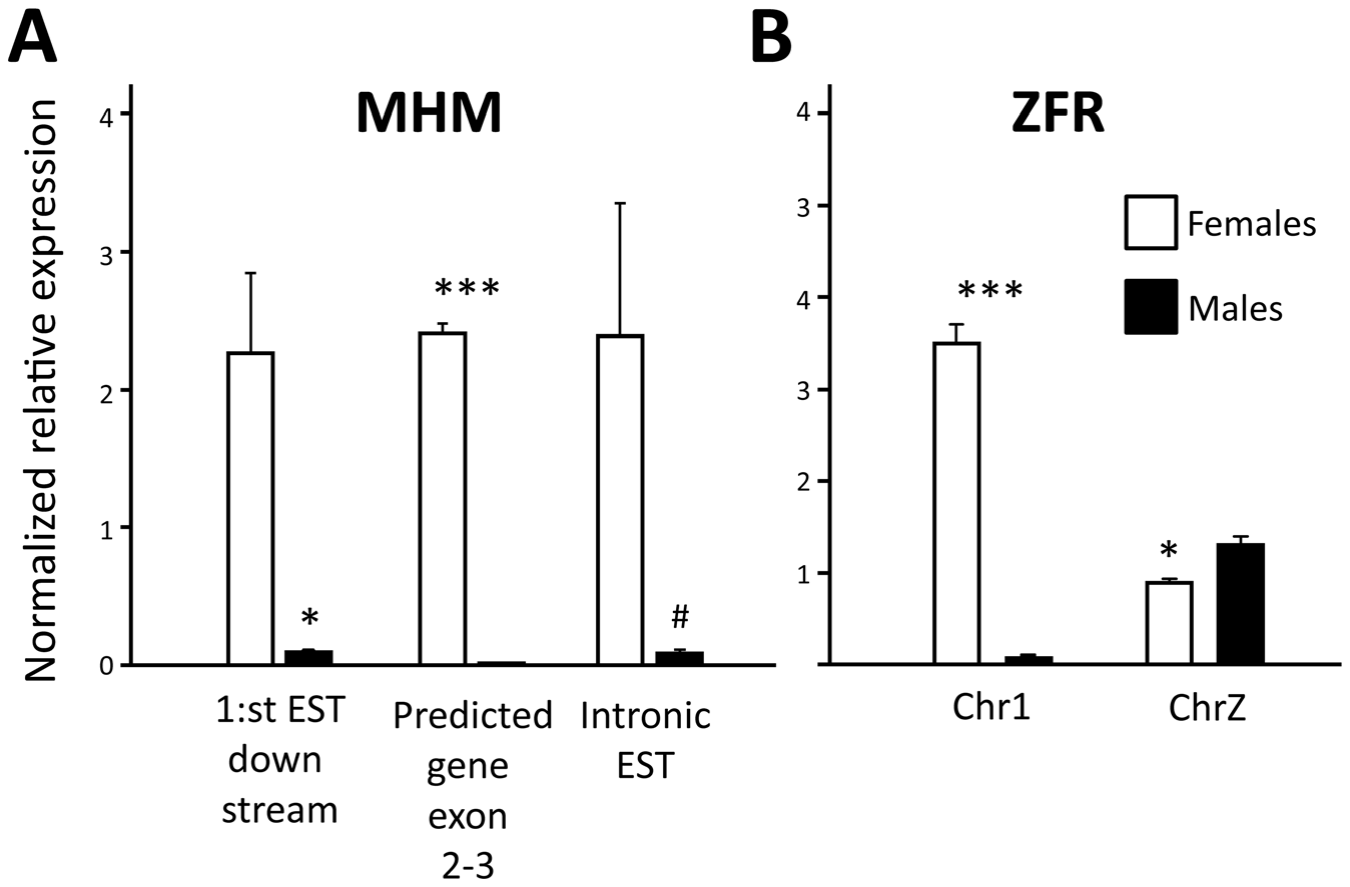
Independent verification of gene expression using qPCR. Gene expression was analyzed on an independent set of RJF brains (females: n = 4, males: n = 7). **A**) The exonic region of the novel gene within MHM was more strongly expressed compared to EST region downstream and within intronic region. **B**) The *ZFR* paralog on chromosome 1 was highly up-regulated in females, while the *ZFR* paralog on chromosome Z showed an expected none dosage compensated male bias. Independent t-test adjusted for unequal variance, ***p<0.0001, *p<0.01, #p<0.1.

For gene expression, the mRNA was hybridized to Affymetrix GeneChip Chicken Genome Arrays. Consistent with previous findings reporting limited dosage compensation in the homogametic avian male, the total proportion of genes on the Z-chromosome that showed lower expression in females was highly significant in both RJF (Figure 1; 87% was lower in females, χ^2^ = 275.8, p<0.0001) and WL (90% was lower in females, χ^2^ = 312.2 p<0.0001) compared to a 50/50 distribution. Significantly differentially expressed (DE) probesets (Table S1) were mainly located on the Z-chromosome and over-represented by lower expression in females in both the RJF (Figure 2A) and WL (Figure 2B). However, most Z linked genes were not significantly DE and, more interestingly, some were strongly up-regulated in females, suggesting active mechanisms against dosage (Figure 3A). Even though three of these genes overlapped with the dosage compensated region surrounding MHM between 25–35 Mb previously identified by Melamed and Arnold [12], most probesets within this region did not reach significant DE. On the contrary, like the rest of the Z chromosome, most probesets within this 10 Mb region had a significantly lower expression in females than in males (RJF: 77% lower in females, χ^2^ = 15.2, p<0.001; WL: 83% lower in females, χ^2^ = 24.4, p<0.0001, compared to a 50/50 distribution) indicating a more complicated control mechanism focused on individual genes than general silencing through mammalian type dosage compensation in the region. One transcript from a novel gene (ENSGALG00000018479) in the periphery of the MHM region on the Z chromosome showed a particularly strong compensational signal (overexpression in females) (Figure 3A). The *DMRT1* gene was not significantly DE between sexes, while the *DMRT3* gene, which is located very close downstream, was so, but only in RJF. To verify and further characterize our results from the MHM region, we performed qPCR on an independent set of RJF brains. This showed that the predicted exonic region of the novel gene in MHM, was strongly DE between sexes, while an intronic EST and a flanking EST region closer to the DMRT1/3 cluster showed much more variation (Figure 4A).

### Gene expression: Autosomal sex dimorphic gene expression

Although the autosomes had fewer significantly DE genes in total, most were up-regulated in females (Table S1). Using cluster analysis, we identified three larger clades with unique sex differences in gene expression, consisting of: 1) W chromosome genes, 2) Z chromosome genes and 3) autosomal genes (cluster analysis is represented by the heat maps of Figure 2A, B and D). There was a large overlap of the significantly DE genes between the breeds (Figure 2C), but most autosomal genes were only DE in one of the two breeds (Figure 2A, B and D), leaving only one autosomal gene verified in the overlap: the *zinc finger RNA binding protein* (*ZFR*) on chromosome 1 (Figure 3D). Two highly similar paralogs to the *ZFR* gene are present in the chicken genome; one on chromosome 1 and the other on Z. To find out which of the two paralogs that was responsible for the observed signals, we compared the genomic sequence of each of them to the sequences of the expression array probeset family GgaAffx.25782.x, as well as the Gga.19912.1.S1_at and the Gga.15339.1.S1_s_at probesets; all of which hybridize to both or either of the two paralogs. Significant DE was only found on the four (out of six) probesets most similar to the chromosome 1 paralog, which strongly indicates that the signal came from the *ZFR* on chromosome 1 (Figure S1). To clarify this further we performed qPCR on our independent set of RJF brains, which showed that the *ZRF* on chromosome 1 was strongly up-regulated in females, while the chromosome Z paralog was instead modestly up-regulated in males, as could be expected under no dosage compensation (Figure 4B).

### DNA-methylation: No mammalian type dosage compensation

**Figure 5:**
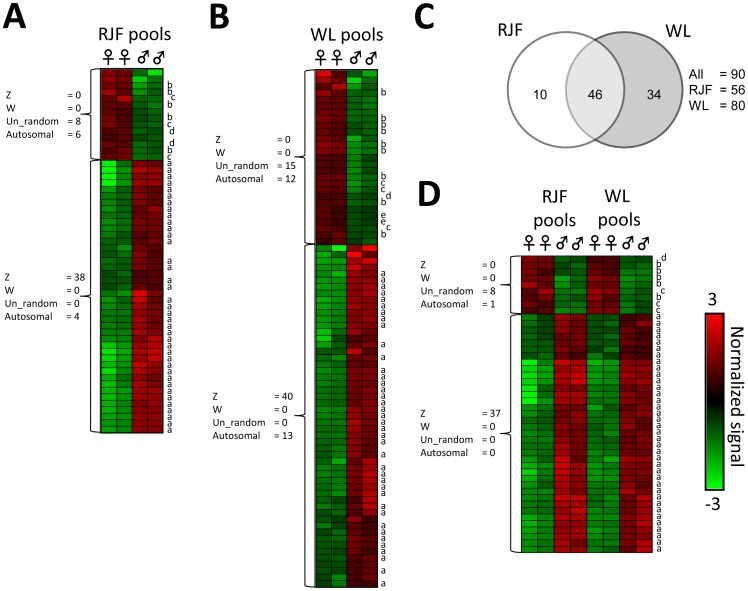
Significant promoter DNA-methylation differences between sexes within and across breeds. Heat map columns represent individual tiling arrays, while rows represent significantly differentially methylated (DM) probes adjusted for false discovery rate. Heat maps are organized using hierarchical cluster analysis (average linkage) identifying two major clades both within and across breeds (represented by brackets). **A**) Probes DM within Red Junglefowl (RJF pools). **B**) Probes DM within White Leghorn (WL pools). **C**) Venn-diagram showing how many of the DM probes within breeds that are present across, in both, breeds. **D**) Probes DM across breeds. Lower case letters next to heat map rows indicate that multiple probes are significantly DM in the same promoter; the same letter indicates the same promoter. Note that “d” and “a” represent probes in the promoters of *ZFR* and the novel gene in MHM respectively. Each tiling array was hybridized with a pool of six birds.

The DNA extracted from the same brain samples as the mRNA was analyzed for methylation differences, using a custom-made tiling array together with methylated DNA immunoprecipitation (MeDIP), which allowed us to analyze 3623 promoter regions (defined as 7.5 kb upstream and 3.5 kb downstream from gene start site) covering about 385K probes simultaneously. Twelve gene promoters in the RJF (Figure 5A) and 31 in WL (Figure 5B) contained significantly differentially methylated (DM) probes between sexes (for details see Table S2). After considering just the overlap between breeds (Figure 5C) four gene promoters remained (Figure 5D).

**Figure 6:**
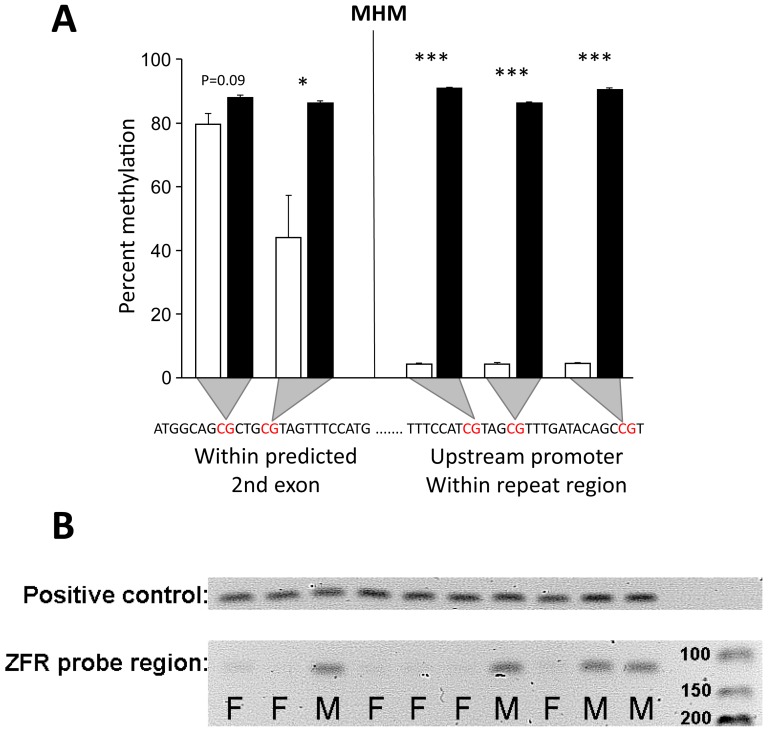
Independent verification of DNA-methylation using bisulfite sequencing. **A**) Bisulfite sequencing of the novel gene within the MHM region shows that the predicted second exon and the repeat region in the promoter have different DNA-methylation patterns. **B**) Agarose gel electrophoresis shows that PCR using bisulfite converted primers annealing in regions with no CG-dinucleotides, failed to amplify in females, but not in males. Independent t-test adjusted for unequal variance, ***p<0.0001, *p<0.01, #p<0.1.

The most differentially methylated region was found in the putative promoter of the novel gene in MHM that also showed the greatest differential expression on the Z chromosome (Figure 3B). There were 37 significantly DM probes hypermethylated in males and all were specifically associated with the promoter of the novel gene within the MHM, while the neighboring genes were unaffected (Figure 3C). Together with the gene expression data, this strongly suggests that sex specific regulation within the MHM region is localized to specific transcripts. To further explore this we performed bisulfite sequencing on our independent set of RJF brains. Interestingly, while a repeat region up-stream of the transcription start site was verified as strongly methylated in males and lacking methylation in females, CpGs within the predicted second exon of the novel gene were methylated in both sexes (Figure 6A).

### DNA-methylation: Autosomal genes and the Zinc finger RNA binding protein

Since *ZFR* was the only autosomal gene verified as significantly DE in both breeds, differential regulation of autosomal genes may mainly contribute to breed specific sex differences, and not sex differences present in both breeds. In line with this, even though plenty of autosomal promoters were significantly DM within breeds (Figure 5A and B), only the promoter to the *ZFR* gene was DM in both breeds (Figure 5D). While male promoter hypermethylation of the novel gene in MHM (Figure 3C) was associated with lower gene expression compared to females (Figure 3B), the opposite relationship was seen on *ZFR* (Figure 3D and E). Notably, the strongly differentially methylated MHM promoter on Z (Figure 3C) contained a CpG island, which the *ZFR* promoter lacked (Figure 3F).

To confidently assign the DM signal to the chromosome 1 paralog of *ZFR* (see previous discussion), we blasted the oligonucleotide sequences of the four strongest DM probes to the chicken genome. While all four perfectly aligned to the promoter region near the chromosome 1 paralog, none aligned to the Z counterpart. We also verified this by bisulfite sequencing on our independent set of RJF brains, using PCR primers specific for the DM region on chromosome 1 after bisulfite conversion. Surprisingly, very little PCR product was generated from female bisulfite treated DNA compared to male DNA (Figure 6B). Since the PCR-primers were designed to not overlap CG-dinucleotides, female cytosine residues at the primer annealing sites might have been protected against bisulfite conversion by another type of DNA-modification than CpG-methylation.

### Functions of differentially expressed or methylated genes

Since our study focused on gene expression and DNA-methylation differences in the brain, we specifically looked for gene candidates with functions in behavioral control. We therefore combined manual annotation with gene ontology analysis, and produced a list of possible “behavioral” genes (Table 1). While none of the genes were both significantly differentially expressed and methylated, many of them were verified between breeds, even though more were present in RJF than in WL. One interesting example was seen in the Tyrosine hydroxylase pathway, which involves dopamine and norepinephrine synthesis, where three key enzymes (*DBH*, *MOXD1*, *TH*) had genes with hypomethylated promoters in RJF females compared to males, while such a relationship was absent in WL. The tyrosine hydroxylase pathway and dopaminergic reward system has repeatedly been associated with different aspects of social and exploratory behavior (for examples see [20], [21] [22]).Furthermore, the two receptor genes *GABRA2* (a GABA receptor subunit) and *NTRK2* (coding for the BDNF TrkB receptor) also showed differential expression/methylation, and both have previously been associated with the reward system [23] [24].

**Table 1:** Top candidate genes related to sexually dimorphic behavior in chickens (Red Junglefowl).

*Ensemble ID*	*Name*	*Function*	*Behavioral keywords*	*Female vs. Male*
*Differentially expressed*				
ENSGALG00000002289	*CHAT*	Cholinergic regulation	Sexual, Learning, Locomotion	Up-regulated
ENSGALG00000004364	*ABR*	Synaptic plasticity	Stress, Learning	Up-regulated
ENSGALG00000016554	*ACE2*	Angiotensin regulation	Feeding, Drinking, Stress	Up-regulated
ENSGALG00000012594	*NTRK2*	Brain neurotrophic factor signaling, Dopamine interaction	Cognition, Learning, Feeding, Fear	Down-regulated
ENSGALG00000014994	*CRHBP*	Regulation of HPA-axis, Dopamine and Glutamate interaction	Stress, Learning, Memory	Down-regulated a
ENSGALG00000015147	*ALDH1A1*	Interaction with Dopamine and Norephrenaphrine pathways	Reward, Learning	Down-regulated a
*Differentially methylated*				
ENSGALG00000014206	*GABRA2*	GABA regulation	Behavior, Reward	Hyper-methylated a
ENSGALG00000007839	*NCAM1*	Synaptic plasticity	Brain development, Learning	Hyper-methylated ^ab^
ENSGALG00000002020	*GLUD2*	Glutamate regulation	Fear, Learning, Memory	Hypo-methylated ^ab^
ENSGALG00000002911	*DBH*	Tyrosine metabolism, Dopamine and Norepinephrine regulation	Reward, Learning, Maternal, Social	Hypo-methylated b
ENSGALG00000002926	*MOXD1*	Tyrosine metabolism, Dopamine and Norepinephrine regulation	Reward, Learning, Maternal, Social	Hypo-methylated b
ENSGALG00000006551	*TH*	Tyrosine metabolism, Dopamine and Norepinephrine regulation	Reward, Learning, Sexual, Social	Hypo-methylated b
ENSGALG00000008875	*PLCB1*	Long term potentiation	Sexual, Cognition, Learning	Hypo-methylated ^ab^
ENSGALG00000015177	*GNA11*	Dopaminergic, Colinergic and Glutamatergic receptor signaling	Reward, Fear, Learning	Hypo-methylated a

a Validated as significant or top ranked differentially expressed or methylated in the White Leghorn breed.

b Not significant, but top ranked among the 225 (of 3623) most differentially methylated promoters.

Another interesting Z-linked gene, which was less expressed in females of both breeds, was the highly conserved *CRHBP* (*corticotrophin releasing hormone binding protein*). In vertebrates, CRH (corticotropin-releasing hormone) is one of the main controllers of the HPA-axis. CRHBP binds CRH and inhibits its interaction with the CRH receptors, thereby working as an anxiolytic agent [25]. Hypothetically, the lower dosage of *CRHBP* expression in female birds would suggest that females may have a stronger stress response. While *CRHBP* is conserved on the Z chromosome in chicken, turkey and zebra finch, but found on many different autosomes in other vertebrates, it may play a particularly important role in behavioral sex differences of birds.

When considering all significant DE genes, GO analysis (Table S3) did not reveal significant terms associated with the biological functions of reproduction and sex development. When only considering the genes up-regulated, in females terms like “sex differentiation” and “external genitalia morphogenesis” were significant. This was mainly due to two paralogs of the *Nipped-B* (*NIPL*) gene on the Z and W chromosomes respectively which were up-regulated in females of both breeds. Similarly, when considering all significant DM promoters (Table S4), no significant terms associated with biological function of reproduction or sex development were found. Nevertheless, disregarding correction for multiple testing (FDR), some interesting terms were high ranked on the lists when looking at WL separately, such as “male, female gonadal development” and “gamete generation”, which was associated mainly with four significantly DM genes: *WNT4, SGPL1*, *CRTAP*, *BCL2*. Interestingly, even though *WNT4* and *CRTAP* were not significantly DM in the RJF, they were still high ranked (531 and 85 out of 383385 probes).

### Behavioral sex differences in Red Junglefowl

Since most of our behavioral gene candidates were identified within the RJF, we subjected 158 males and 141 females of this breed to a battery of standardized behavioral tests designed mainly to phenotype different aspects of fear, exploration and sociality applied at different ages. We hypothesized that females should express more fear related behaviors than males based on a higher expression of *CRHBP* in males, and that the sexes should differ in novelty exploration and sociality, since this previously was shown to be affected by the tyrosine hydroxylase pathway and dopamine release (see previous discussion).

The behavioral test results are all summarized in Table S5. Social affiliation and companion propensity was tested in a social reinstatement test at three weeks of age, but did not detect any significant differences between the sexes. In an open field test one week later, males entered the center zone quicker and more often than females, and spent less time in the corners of the arena, which all indicates less fear in males. No differences were detected when we repeated the test at 16 weeks of age. At 13 weeks a foraging and exploration test was conducted where males were significantly less prone to explore the arena and to exploit novel and hidden food. To further measure fearfulness, we used two different tests, where the first measured the reactions to a simulated aerial predator attack, and the second was the widely used tonic immobility (TI) test. These tests were applied when the birds were 15 and 17 weeks old respectively. Females preened less, vocalized more and made more escape attempts in predator test, while they required more induction attempts and remained longer in TI; all indicates more fear in females. At 27 weeks, undisturbed behavior was recorded while groups of birds were roaming freely in an enriched pen. Here, females explored more and were more active, while males performed more perching, comfort and jump/flight behavior.

In general, the tests suggest that females were more explorative and prone to forage, but also more fearful in their reactions to various stressful stimuli. To derive more general reaction patterns that may differ between the sexes, we used a two-step principal component analysis (PCA). First, we entered all behavioral variables from all the tests (see Table S5) in a PCA, which generated four factors with an eigenvalue over one. From this first solution, we selected one variable from each test (the one with the highest communality in the four-factor solution) and performed a second PCA based on these seven variables. The results of the second PCA, rendering four factors that explained 71% of the variation, are shown in Table 2. The first factor mainly spanned SR-social and UB-explore, and was tentatively named “Social/Exploration”. The second factor was named “Fear” since TI-first had the highest loading. The interpretation of the third factor was more elusive, but based on the observed loadings was tentatively labeled “Activity”. The last factor was labeled “Foraging/Exploration”.

**Table 2:** Factor scores from principal component analysis (PCA) of behavioral variables with the highest communality measured in a series of tests on Red Junglefowls.*

	Factors			
Variables	1	2	3	4
SR duration social zone (s)	−0.81	0.07	0.31	−0.12
OF4 frequency center	−0.35	−0.33	−0.65	0.33
FE Hidden food	0.26	0.50	0.26	0.59
AP Explore	0.43	−0.38	0.40	−0.34
OF16 crossed zones	−0.19	−0.50	0.45	0.56
TI First movement	−0.10	0.70	0.00	−0.03
UB Explore	0.85	−0.07	−0.15	0.13

Test abbreviation: SR =  Social reinstatement, OF4 =  Openfield at 4 weeks of age, FE =  Foraging and exploration, AP =  Aerial predator, OF16 = Openfield at 16 weeks of age, TI =  Tonic immobility, UB =  Undisturbed behavior.

*For all behavior test results see Table S3.

We then calculated factor scores for all individuals on each of the four factors, and tested for sex differences. As seen in Table 3, there were significant sex differences on the three first factors. Females scored higher on all of them, indicating higher exploration, fear and general activity, which supports the findings from the genetic and epigenetic analysis discussed above.

**Table 3:** Mean factor scores for the four factors obtained in the between-test PCA, and the P-value obtained from a t-test of the difference between the sexes.

		Females		Males		
PCA Factor	Interpretation	Mean	SEM	Mean	SEM	p-value
1	Social/Exploration	0.19	±0.09	−0.20	±0.09	0.002
2	Fear	0.23	±0.09	−0.24	±0.09	0.0002
3	Activity	0.15	±0.09	−0.16	±0.08	0.02
4	Foraging/Exploration	−0.01	±0.09	0.01	±0.09	0.9

### General discussion

Our data show consistent differences in behavior and genome wide gene expression, and to a smaller extent, DNA-methylation between sexes in chickens. As previously reported, it is clear that the juvenile chicken brain has very limited mammalian type dosage compensation, and we show that this also account for DNA-methylation on the Z-chromosome. In fact, few sites are strongly differentially methylated in promoter regions between sexes in chickens. One of these sites, the so called male hyper-methylated region (MHM) [14] on the Z-chromosome, is linked to a large methylated sequence, which selectively suppresses expression of a specific transcript in males. We also found a highly methylated site associated with female up-regulation of the *zinc finger RNA binding protein* gene on chromosome 1, which still is functionally uncharacterized. Seemingly, this site was also associated with non-CpG-methylation (see further discussion below). Additionally, we identified several differentially expressed genes known to affect behavior, and as suggested from their functional annotation, we showed that female Red Junglefowl are more explorative and fearful.

Teranishi *et al.* showed that a cloned 2.2 kb repetitive sequence on the short arm of the Z chromosomes is strongly hypermethylated in male chickens (the MHM region) and mainly transcribes a long non-coding RNA in females [14]. We are aware that the DE/DM signal in the MHM region in the present study could originate from the same ncRNA. Nevertheless, the MHM contains several open reading frames which could potentially translate into proteins [26]. In addition, within the MHM region there is a predicted gene with three possible exons, where the two last is located within a region with no repeats (Ensembl genebuild). Since the DE signal is associated only with the 1.8 kb probeset covering these exons and not the neighboring probesets, spanning other parts of the 9.5 kb ncRNA [14], our signal could still come from a protein coding gene or a splice variant of the ncRNA. Furthermore, while Teranishi *et al.* [14] originally found MHM and its associated long ncRNA in various tissues and developmental stages, analysis of chicken embryos using the Affymetrix's GeneChip Chicken Genome array has in some cases failed to detected differential expression of this probeset [27], [28]. Since other studies, including our own, report very strong female bias using the same platform but at other developmental stages, tissues and genetic backgrounds [13], [29], it suggests dynamic regulation of the MHM region.

Manks and Ellegren [13] have concluded that dosage compensation in chickens is regulated on a gene-by-gene level. Our qPCR and bisulfite sequencing analysis adds another level of complexity to the regulation of the MHM region by showing that the exonic part of the novel MHM gene shows much less variation between sexes than surrounding regions. The impact of this regional difference is still unknown, but it is tempting to speculate that the region may harbor a dynamic transcription pattern, involving both non-coding and coding RNA important for sex differentiation. Interestingly, Bisoni *et al.* have also associated the MHM region with female histone H4 hyper-acetylation [30], an epigenetic mark usually associated with activation of transcription. It remains to be investigated how specific this histone modification is targeted to the exonic region of novel gene in MHM, but since Bisoni *et al.* did not find differential H4 acetylation on the adjacent *DMRT1* gene, there may be a relationship between female H4 acetylation and female DNA(hypo)-methylation at this very specific site.

In humans, the loss of one copy of *DMRT1* can cause phenotypic sex reversal [16], and has been implicated as important also in bird sex determination [15]. Notably, *DMRT1* is located very close to the MHM region. In our study, *DMRT1* transcripts were not significantly DE between sexes, as they are in chicken embryos [26], [27]. Instead we found that the first exon of *DMRT3*, a paralog located only 4.5 kb downstream from *DMRT1*, was strongly down-regulated in RJF males. Perhaps *DMRT1* and *DMRT3* play different roles in sexual development at different ages and tissues. Interestingly, we found no down-regulation of *DMRT3* in the WL breed. Due to commercial selection, the WL breed becomes sexually mature weeks before the RJF, which again may suggest that *DMRT1/3* expression is age dependent.

A few probesets of the *ZFR* gene on chromosome 1 have previously been found to have a female expression bias in chickens [29] [28]. While little is known about the function of this *ZFR* paralog, from its RNA binding properties it seems plausible that it plays an important role in RNA regulation during sexual development. Future experiments should therefore investigate if there are interactions between ZFR and the long non-coding RNA in the MHM region. The *ZFR* promoter is relatively CG poor and our results suggest that it contains non-CG-dinucleotide DNA-modifications. Methylation at CHG and CHH (where H can be either A, T or G) is commonly seen in plants, but does not occur widely in mammals [31]. To our knowledge this is the first time an indication of such a modification has been reported in an avian species, but it is clear that it needs further investigation. We also see an unexpected up-regulation of *ZFR* despite hypermethylation in females, which may indicate that a novel kind of epigenetic regulatory mechanism that are CG independent, or depend on both CG- and non-CG-sequence combinations, might be present in the region. Furthermore, we have previously demonstrated that a QTL covering the region around *ZFR* explains about 23% and 15% of the two fold differences in body weight between RJF and WL, in males and females respectively [32]. This QTL has also been associated with differences in fearfulness as measured by the tonic immobility test [33]. The *ZRF* gene has recently been suggested to be one of the most promising causal candidates within this QTL [34], but its precise functions remain to be investigated.

A large part of significantly DE and DM genes/promoters was associated with the un_random chromosome, which is a collection of unlinked sequences still awaiting alignment to real chromosomes. Since these genes lie randomly in relation to each other we decided to disregard them in many of our downstream analyses. However, after careful examination of our cluster analysis it seems likely that many of the significant un_random genes are in fact located on the sex chromosomes.

We initially designed our DNA-methylation tiling array to detect differences generated during chicken domestication, which might have biased the regions under analysis towards those important for this biological process. Nevertheless, making a gene ontology analysis of the 3623 gene promoters on the array did not show a significant enrichment of specific biological processes, which suggests an unbiased representation.

Many previous studies have explored sex differences in behavior, in many different species. For example, differences in foraging and social behavior, as well as stress and anxiety levels has widely been demonstrated in the wild [35] [36] [37] and under experimental conditions [38] [39]. Genetic findings of the present study suggest that sex behavioral differences are ubiquitous also in chickens. The battery of tests applied on the Red Junglefowl verified this, and across tests, females were more explorative, fearful and active. In the wild, Red Junglefowl form small harem-groups with one dominant male, a few subdominant males and a couple of females [18]. In such a group, the sexes have different roles and behaviors, where male reproductive success is more related to dominance and female success depends on maternal care. For example, high-ranked males have more access to females, provides females with resources such as food (courtship feeding) [40] [41] and defends the territory more vigilantly than low ranked males [42]. Females on the other hand incubate eggs, raise offspring without help from the males and do not participate in territory defense [43] [44]. Hence, one might expect that selection has favored different traits in the two sexes. Our results confirm these expected behavioral differences, and provide, for the first time a list of genomic sites associated with variation in gene expression and DNA-methylation that may be important for their development.

## Conclusion

We have identified hundreds of genes genome wide with differential expression between sexes, but few differences in promoter DNA-methylation. After probing hundreds of promoters on the Z-chromosome, we can now for the first time be confident in saying that juvenile chicken lack mammalian type dosage compensation associated with large scale promoter DNA-methylation differences between sexes. On the other hand, our results indicate another type of cytosine-modification independent of CpG-methylation, which may play an important role in avian gene regulation and therefore stresses further investigation. Our results are important not only to understand the fundamental differences in how birds and mammals cope with a sexually unbalanced gene dosage, but also to understand how epigenetic factors may shape morphological, physiological as well as behavioral sex differences. Although the present results do not allow us to infer causal relationships between the epigenetic variation and the specific behavioral differences, the strong correlations call for further studies to reveal such connections.

## Methods

### Ethics statement

The experiments were approved by the “Regional Committee for Ethical Approval of Animal Experiments” in Linköping, Sweden (permit no 122-10). During all experiments, animals were handled carefully and in such a way that unnecessary stress was avoided. Animals were moved and transported in darkness, which reduces the stress responses of chickens, and were never exposed to any physical harmful stimuli. Killing was done by rapid dislocation of the necks, according to prevailing welfare standards and the ethical permit.

### Breeding and housing

All birds in the experiment were pedigree bred and hatched. The eggs were incubated at 37.5°C and 55% relative humidity, with egg rotation every hour in a Marsalles 25 DIGIT incubator. At day 17 the temperature was increased to 37.8°C, the humidity to 65% and rotation turned off. The first day after hatching, birds were wing-tagged, weighed and vaccinated against Marek's disease. In the hatchery unit (“Kruijt”) at Linköping University, the animals were held in small pens (0.75x0.75 m) in mixed-sex groups of about 30 birds with feed/water ad libitum and wood chip flooring. Pens were enlarged to double the size after two weeks and furnished with perches. Room temperature was about 27°C.

At five weeks of age animals were moved to a chicken research facility (“Wood-Gush”) situated 10 km from the university, where they were kept in pens measuring 3x3x3 m and in sex separated groups of about 40 birds. The pens had a three-floor system with perches, nest boxes, and wood chips on the floor. Food and water was provided *ad libitum*.

### Gene expression and DNA-methylation microarrays

Since the DNA-methylation tiling array was originally designed using WASHUC2 all results are presented using this genebuild's coordinates. For conversion to GalGal4 visit http://www.ensembl.org/Gallus_gallus/Info/Index.

Animal material, tissue sampling, preprocessing and micro/tiling array hybridization have been described in detail elsewhere [45]. In brief, breeds used for the gene expression and DNA methylation analysis originated out of 1) an outbreed zoo population of Red Junglefowl (RJF) kept as an experimental line for ten generations, and 2) a domesticated White Leghorn (WL) egg layer used as an experimental line since the 1970's.

In both breeds, brain samples were taken from 21 days old chicks (12 birds of each sex). From each brain, a region containing thalamus and hypothalamus was dissected out, immediately frozen and later homogenized in TRI reagents. RNA was isolated using the TRI manufacturer protocol with additional RNeasy (Qiagen) clean up, while DNA was isolated from the same TRI homogenate by ethanol precipitation followed by DNeasy (Qiagen) clean up. Before further processing, both RNA and DNA samples were separately mixed in eight pools with six brains per pool (two replicates of each sex within breed, in total 24 brains of each sex). For gene expression analysis, the RNA-pools were labeled and hybridized to the GeneChip Chicken Genome Array (Affymetrix) capable of analyzing 33457 transcripts. For DNA-methylation analysis, the DNA-pools were split, enriched using methylated DNA immunoprecipitation (MeDIP) and later co-hybridized with the original sample to a custom made DNA-methylation tiling array (Roche-NimbleGen) containing tiled 385K probes from 3623 promoters (defined as 7.5 kb upstream and 3.5 kb downstream from gene start site). The array data has been deposited at ArrayExpress and can be accessed with the following accession numbers: Gene expression arrays [E-MTAB644]; DNA-methylation [EMTAB-649].

### Verification of microarray data

Hypothalamus tissue was collected and RNA/DNA extraction was done in same way as for the microarray experiment, but taken from three weeks old Red Junglefowls of an independent experimental population maintained in our lab. For gene expression verification, 1 µg RNA was reversely transcribed to cDNA using the RevertAid First Strand cDNA synthesis kit (Fermentas/Thermo Scientific) with oligo-dT primers and qPCR was performed with transcript specific primers seen in Table S6 using a 55°C annealing temperature. For DNA-methylation verification, 600 ng DNA was bisulfite converted using the EpiTect Fast Bisulfite Conversion Kit (Qiagen), amplified through PCR using the PyroMark PCR kit (Qiagen) and sequenced on a PyroMark 96 MD pyrosequencer. Prior to sequencing, a single PCR product was verified by 2% agarose gel electrophoresis. Bisulfite converted PCR and sequencing primers were designed using the PyroMark Assay Design software (Qiagen) and can be found in Table S6. PCR was performed at 94°C for 30 sec, 56°C for 30 sec and 72°C for 30 sec, through 45 cycles. All procedures followed manufactures recommendations.

### Behavioral tests

Behavior was recorded in a Red Junglefowl population described previously by [46] and the behavioral tests used, except the undisturbed behaviors, were described comprehensively in the same publication. All behavioral variables used in the analysis are presented in Table S5. In brief, we studied 299 birds (158 males and 141 females) from three successive generations, originating from a cross between two captive zoo populations (the Gotala and Copenhagen population; the former being the RJF population used in the micro/tiling array experiments). Sociality was tested at three weeks of age by measuring duration in the social zone closest to a companion bird and distance moved in a standardized social reinstatement (SR) test (arena  = 20×120×40 cm, social zone  = 20×40 cm, test duration  = 5 min, test variables were averaged between two duplicative trails for each animal). Fear/anxiety levels was tested at four (OF4) and sixteen (OF16) weeks of age by measuring frequency in center zone and duration in the peripheral part of an open field arena (arena  = 4 weeks: 80×120×40 cm: 16 weeks: 190×190×100 cm, center zone  = 4 weeks: 40×80 cm; 16 weeks: 100×100×100 cm, test duration: 5 min, test variables were averaged between two duplicative trails for each animal and age). Foraging and explorative behaviors were recorded at 13 weeks of age by measuring number of pecks directed at a bowl with hidden food (meal worms in wood shavings) in a choice between two other potential food resources (only wood shavings and familiar food pellets) and the frequency of changing between four identical clusters of food bowls, each containing all three food resources (arena: 95×95 cm, food deprivation: 1 h before trial, habituation to arena: 10 min before trail, test duration: 5 min) Anti-predator reaction (AP) was recorded at 15 weeks of age by measuring preening, vocalization, freezing and escape attempts after a brief exposure to an aerial predator model (hawk silhouette)(arena: 50×150×50 cm, baseline pre-test before predator: 5 min, test duration after predator: 5 min). General fearfulness, as measured by the tonic immobility reaction (TI), was recorded at 17 weeks of age by measuring number of induction attempts until the birds entered tonic immobility, as well as the time to first head movement and the duration of immobility (test duration: 10 min; if birds were immobile for more, a maximum value of 600 sec was given).

Undisturbed behaviors (UB) were recorded at 27 weeks of age in a pen similar to the home pen, measuring 2.5x3.0x3.0 m and furnished with a perch, wood chip flooring and *ad libitum* food and water. Habituation took place in groups of 7–11 familiar individuals (same sex) from 3 p.m. the day before observations began. At 9 a.m. behavioral observations started, using one-zero sampling with 10 seconds intervals and rotating focal animal sampling with five minutes for each individual. Recordings were repeated twice between 9-12 a.m. and twice between 2–5 p.m. Each animal was observed for a total of 20 minutes and percentage of exploration, activity, perching, jump/flight and comfort behavior was recorded.

### Statistical analyses

Gene expression microarrays were normalized, filtered and analyzed with R/Bioconductor (www.bioconductor.org) using the affyPLM, geneFilter and limma packages as in [47]. DNA-methylation tiling arrays were analyzed using the Ringo, geneFilter and limma packages as in [45]. All array results were adjusted for multiple testing using false discovery rate (FDR, BH) algorithms [48]. The gene expression dataset was filtered to remove the non-variable and least variable probesets (IQR, cut off at 0.5; [49]). A low number of significantly differentially methylated probes suggested a high number of false negatives in DNA-methylation dataset, filtering up to 80% of the probes did not change the results significantly, hence the data was left unfiltered for downstream analysis. Gene ontology analysis was performed using human ortholog conversion with the Manteia web tool (manteia.igbmc.fr). The Ensembl's genome browser, BLASTN and BioMart tools were used for alignment and annotation analysis using the WASHUC2 genebuild. The Genesis and Statistica software were used for general statistical analysis.

For behavioral variables, qPCR and bisulfite sequencing means and SEM were calculated within sex. Bisulfite sequencing and qPCR data was normally distributed (determined by Shapiro-Wilk test) and analyzed using t-test adjusted for unequal variance (determined by Levene's test). Due to lack of normality the effects of sex on behaviors were assessed through a non-parametric Mann-Whitney U-test. Principal component analysis (PCA) was conducted in order to find patterns of behavior across the different tests. By first entering all variables into the PCA, and from that, choosing the variable from each test with highest communality on a four-factor solution (with eigenvalues over 1.0), we ended up with seven variables which were again entered into a “restricted” PCA. The resulting four factors explained 71% of the variance and the effects of sex on each factor score were then tested using a t-test.

## Supporting Information

Figure S1 **Fold change expression differences between females and males in probesets annotated to both or either of the two ZFR paralogs on chromomse 1 and Z respectively.** Percentages on top of the columns indicate the sequence similarities of each probeset to the chromosome 1 and Z gene paralogs respectively. Note that only the probesets more similar to the chromosome 1 paralogs are significant. ***p<0.001, *p<0.05, #p<0.1 (adjusted for false discovery rate).
(TIF)

Table S1 **Significantly differentially expressed probesets in females related to males.**
(PDF)

Table S2 **Significantly differentially methylated probes in females related to males.**
(PDF)

Table S3 **Gene ontology analysis of significantly differentially expressed (DE) genes in females related to males.**
(PDF)

Table S4 **Gene ontology analysis of significantly differentially methylated (DM) promoters in females related to males.**
(PDF)

Table S5 **Mean values of the behavior variables recorded in each of the behavioral test.**
(PDF)

Table S6 **Primers used in qPCR and bisulfite sequencing.**
(PDF)

## References

[1] Alverdes F (1927)(reprint version 1999)) Social Life in the Animal World. London: Routledge.

[2] Taborsky M, Grantner A (1998) Behavioural time–energy budgets of cooperatively breeding Neolamprologus pulcher (Pisces: Cichlidae). Animal Behaviour 56: 1375–1382. 9933533 10.1006/anbe.1998.0918

[3] Ellegren H, Parsch J (2007) The evolution of sex-biased genes and sex-biased gene expression. Nat Rev Genet 8: 689–698. 17680007 10.1038/nrg2167

[4] Ranz JM, Castillo-Davis CI, Meiklejohn CD, Hartl DL (2003) Sex-Dependent Gene Expression and Evolution of the Drosophila Transcriptome. Science 300: 1742–1745. 12805547 10.1126/science.1085881

[5] Lenz KM, Nugent BM, McCarthy MM (2012) Sexual differentiation of the rodent brain: Dogma and beyond. Frontiers in Neuroscience. 10.3389/fnins.2012.00026 PMC3282918 22363256

[6] Dewing P, Shi T, Horvath S, Vilain E (2003) Sexually dimorphic gene expression in mouse brain precedes gonadal differentiation. Molecular Brain Research 118: 82–90. 14559357 10.1016/s0169-328x(03)00339-5

[7] Lyon MF (1961) Gene Action in the X-chromosome of the Mouse (Mus musculus L.). Nature 190: 372–373. 13764598 10.1038/190372a0

[8] Augui S, Nora EP, Heard E (2011) Regulation of X-chromosome inactivation by the X-inactivation centre. Nat Rev Genet 12: 429–442. 21587299 10.1038/nrg2987

[9] Basu R, Zhang L-F (2011) X chromosome inactivation: A silence that needs to be broken. genesis 49: 821–834. 21898762 10.1002/dvg.20792

[10] Yasukochi Y, Maruyama O, Mahajan MC, Padden C, Euskirchen GM, et al (2010) X chromosome-wide analyses of genomic DNA methylation states and gene expression in male and female neutrophils. Proceedings of the National Academy of Sciences 107: 3704–3709. 10.1073/pnas.0914812107 PMC2840519 20133578

[11] Naurin S, Hansson B, Bensch S, Hasselquist D (2010) Why does dosage compensation differ between XY and ZW taxa? Trends in Genetics 26: 15–20. 19963300 10.1016/j.tig.2009.11.006

[12] Melamed E, Arnold A (2007) Regional differences in dosage compensation on the chicken Z chromosome. Genome Biology 8: R202. 17900367 10.1186/gb-2007-8-9-r202 PMC2375040

[13] Mank JE, Ellegren H (2008) All dosage compensation is local: Gene-by-gene regulation of sex-biased expression on the chicken Z chromosome. Heredity 102: 312–320. 18985062 10.1038/hdy.2008.116

[14] Teranishi M, Shimada Y, Hori T, Nakabayashi O, Kikuchi T, et al (2001) Transcripts of the MHM region on the chicken Z chromosome accumulate as non-coding RNA in the nucleus of female cells adjacent to the DMRT1 locus. Chromosome Research 9: 147–165. 11321370 10.1023/a:1009235120741

[15] Raymond CS, Kettlewell JR, Hirsch B, Bardwell VJ, Zarkower D (1999) Expression of Dmrt1 in the Genital Ridge of Mouse and Chicken Embryos Suggests a Role in Vertebrate Sexual Development. Developmental Biology 215: 208–220. 10545231 10.1006/dbio.1999.9461

[16] Raymond CS, Shamu CE, Shen MM, Seifert KJ, Hirsch B, et al (1998) Evidence for evolutionary conservation of sex-determining genes. Nature 391: 691–695. 9490411 10.1038/35618

[17] Borowicz VA, Graves HB (1986) Social preferences of domestic hens for domestic vs. red junglefowl males and females. Behavioural Processes 12: 125–134. 24897347 10.1016/0376-6357(86)90051-3

[18] Collias NE, Collias EC (1996) Social organization of a red junglefowl, Gallus gallus, population related to evolution theory. Animal Behaviour 51: 1337–1354.

[19] McBride G, Parer IP, Foenander F (1969) The social organization and behaviour of the feral domestic fowl. Animal Behaviour Monographs 2: 127–181.

[20] Cavanaugh BL, Lonstein JS (2010) Social novelty increases tyrosine hydroxylase immunoreactivity in the extended olfactory amygdala of female prairie voles. Physiology and Behavior 100: 381–386. 20381508 10.1016/j.physbeh.2010.03.020 PMC2884985

[21] Tochigi M, Otowa T, Hibino H, Kato C, Otani T, et al (2006) Combined analysis of association between personality traits and three functional polymorphisms in the tyrosine hydroxylase, monoamine oxidase A, and catechol-O-methyltransferase genes. Neuroscience Research 54: 180–185. 16360899 10.1016/j.neures.2005.11.003

[22] Pawlak CR, Ho Y-J, Schwarting RKW (2008) Animal models of human psychopathology based on individual differences in novelty-seeking and anxiety. Neuroscience & Biobehavioral Reviews 32: 1544–1568. 18619487 10.1016/j.neubiorev.2008.06.007

[23] Enoch M-A (2008) The role of GABAA receptors in the development of alcoholism. Pharmacology Biochemistry and Behavior 90: 95–104. 10.1016/j.pbb.2008.03.007 PMC2577853 18440057

[24] Lobo MK, Covington HE III, Chaudhury D, Friedman AK, Sun H, et al (2010) Cell Type-Specific Loss of BDNF Signaling Mimics Optogenetic Control of Cocaine Reward. Science 330: 385–390. 20947769 10.1126/science.1188472 PMC3011229

[25] Westphal NJ, Seasholtz AF (2006) CRH-BP: the regulation and function of a phylogenetically conserved binding protein. Front Biosci 11: 1878–1891. 16368564 10.2741/1931

[26] Roeszler KN, Itman C, Sinclair AH, Smith CA (2012) The long non-coding RNA, MHM, plays a role in chicken embryonic development, including gonadogenesis. Developmental Biology 366: 317–326. 22546690 10.1016/j.ydbio.2012.03.025

[27] Itoh Y, Melamed E, Yang X, Kampf K, Wang S, et al (2007) Dosage compensation is less effective in birds than in mammals. Journal of Biology 6: 2. 17352797 10.1186/jbiol53 PMC2373894

[28] Zhang S, Mathur S, Hattem G, Tassy O, Pourquie O (2010) Sex-dimorphic gene expression and ineffective dosage compensation of Z-linked genes in gastrulating chicken embryos. BMC genomics 11: 13. 20055996 10.1186/1471-2164-11-13 PMC2821371

[29] Lee SI, Lee WK, Shin JH, Han BK, Moon S, et al (2009) Sexually dimorphic gene expression in the chick brain before gonadal differentiation. Poultry science 88: 1003–1015. 10.3382/ps.2008-00197 19359689

[30] Bisoni L, Batlle-Morera L, Bird AP, Suzuki M, McQueen HA (2005) Female-specific hyperacetylation of histone H4 in the chicken Z chromosome. Chromosome Research 13: 205–214. 15861309 10.1007/s10577-005-1505-4

[31] He X-J, Chen T, Zhu J-K (2011) Regulation and function of DNA methylation in plants and animals. Cell Res 21: 442–465. 21321601 10.1038/cr.2011.23 PMC3152208

[32] Kerje S, Carlborg Ö, Jacobsson L, Schütz K, Hartmann C, et al (2003) The twofold difference in adult size between the red junglefowl and White Leghorn chickens is largely explained by a limited number of QTLs. Animal Genetics 34: 264–274. 12873214 10.1046/j.1365-2052.2003.01000.x

[33] Schütz KE, Kerje S, Jacobsson L, Forkman B, Carlborg Ö, et al (2004) Major Growth QTLs in Fowl Are Related to Fearful Behavior: Possible Genetic Links between Fear Responses and Production Traits in a Red Junglefowl x White Leghorn Intercross. Behavior Genetics 34: 121–130. 14739702 10.1023/B:BEGE.0000009481.98336.fc

[34] Crooks L, Carlborg Ö, Marklund S, Johansson AM (2013) Identification of Null Alleles and Deletions from SNP Genotypes for an Intercross Between Domestic and Wild Chickens. G3: Genes|Genomes|Genetics. 10.1534/g3.113.006643 PMC3737165 23708300

[35] Boyd LE (1988) Time budgets of adult Przewalski horses: Effects of sex, reproductive status and enclosure. Applied Animal Behaviour Science 21: 19–39.

[36] Hoffman SG (1983) Sex-related foraging behavior in sequentially hermaphroditic hogfishes (Bodianus spp.). Ecology 64: 798–808.

[37] McKeegan DEF, Deeming DC (1997) Effects of gender and group size on the time-activity budgets of adult breeding ostriches (Struthio camelus) in a farming environment. Applied Animal Behaviour Science 51: 159–177.

[38] Johnston AL, File SE (1991) Sex differences in animal tests of anxiety. Physiology and Behavior 49: 245–250. 2062894 10.1016/0031-9384(91)90039-q

[39] Palanza P, Gioiosa L, Parmigiani S (2001) Social stress in mice: Gender differences and effects of estrous cycle and social dominance. Physiology and Behavior 73: 411–420. 11438369 10.1016/s0031-9384(01)00494-2

[40] Evans CS, Evans L (1999) Chicken food calls are functionally referential. Animal Behaviour 58: 307–319. 10458882 10.1006/anbe.1999.1143

[41] Kruijt JP (1966) The Development of Ritualized Displays in Junglefowl. Philosophical Transactions of the Royal Society of London Series B, Biological Sciences 251: 479–484.

[42] Johnson RA (1963) Habitat Preference and Behavior of Breeding Jungle Fowl in Central Western Thailand. The Wilson Bulletin 75: 270–272.

[43] Collias NE, Collias EC (1967) A Field Study of the Red Jungle Fowl in North-Central India. The Condor 69: 360–386.

[44] Sullivan MS (1991) Flock structure in red junglefowl. Applied Animal Behaviour Science 30: 381–386.

[45] Natt D, Rubin C-J, Wright D, Johnsson M, Belteky J, et al (2012) Heritable genome-wide variation of gene expression and promoter methylation between wild and domesticated chickens. BMC genomics 13: 59. 22305654 10.1186/1471-2164-13-59 PMC3297523

[46] Agnvall B, Jöngren M, Strandberg E, Jensen P (2012) Heritability and Genetic Correlations of Fear-Related Behaviour in Red Junglefowl–Possible Implications for Early Domestication. PLoS One 7: e35162. 22536354 10.1371/journal.pone.0035162 PMC3334967

[47] Goerlich VC, Nätt D, Elfwing M, Macdonald B, Jensen P (2012) Transgenerational effects of early experience on behavioral, hormonal and gene expression responses to acute stress in the precocial chicken. Hormones and behavior 61: 711–718. 22465454 10.1016/j.yhbeh.2012.03.006

[48] Benjamini Y, Hochberg Y (1995) Controlling the False Discovery Rate: A Practical and Powerful Approach to Multiple Testing. Journal of the Royal Statistical Society Series B (Methodological) 57: 289–300.

[49] Bourgon R, Gentleman R, Huber W (2010) Independent filtering increases detection power for high-throughput experiments. Proceedings of the National Academy of Sciences 107: 9546–9551. 10.1073/pnas.0914005107 PMC2906865 20460310

