# Supplementary figures and images for: Large Sex Differences in Chicken Behavior and Brain Gene Expression Coincide with Few Differences in Promoter DNA-Methylation
Source: PLoS One. 2014 Apr 29;9(4):e96376. doi: 10.1371/journal.pone.0096376 (PMC4004567; doi:10.1371/journal.pone.0096376)

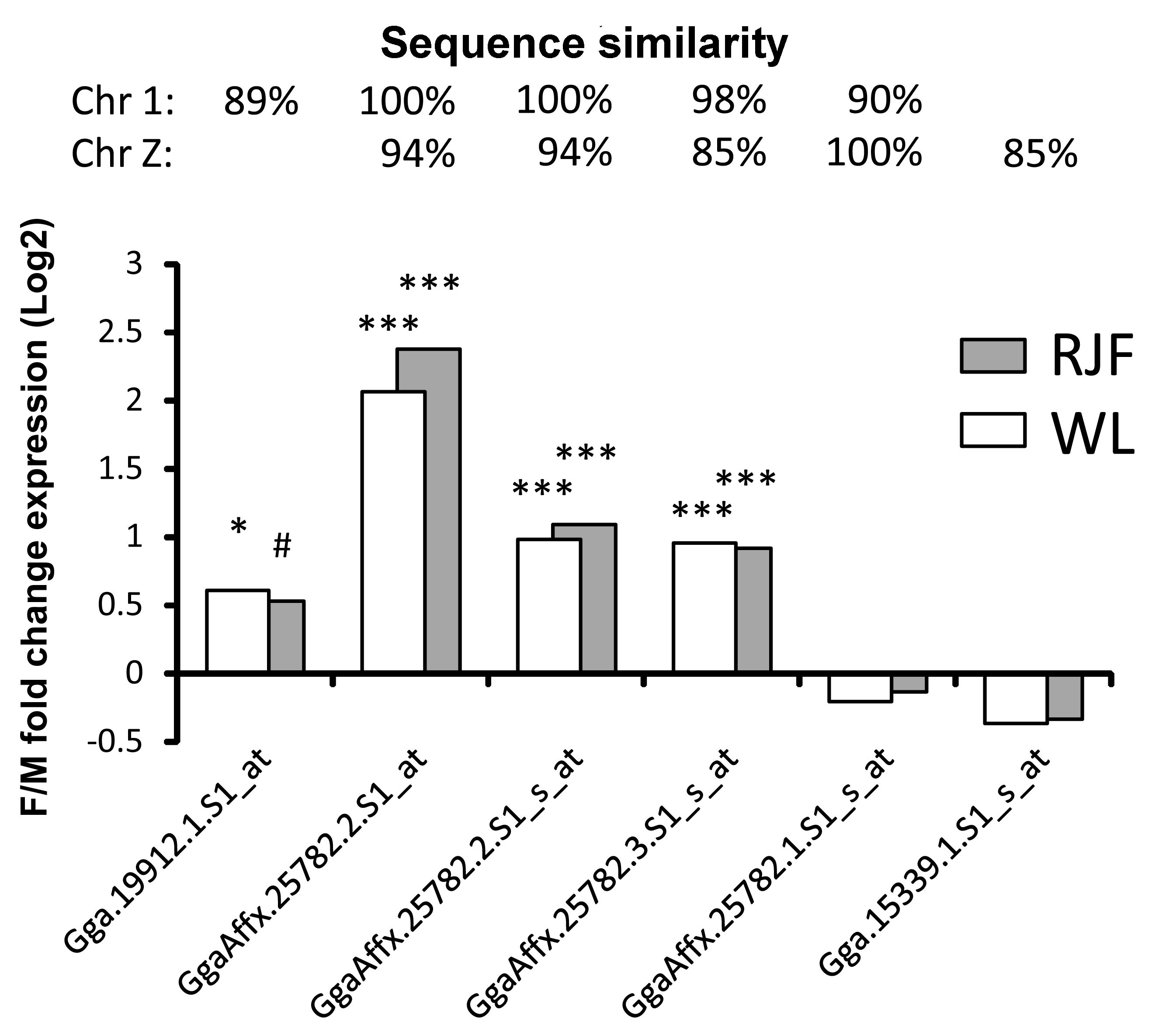

Supplement: Figure S1 — Fold change expression differences between females and males in probesets annotated to both or either of the two ZFR paralogs on chromomse 1 and Z respectively. Percentages on top of the columns indicate the sequence similarities of each probeset to the chromosome 1 and Z gene paralogs respectively. Note that only the probesets more similar to the chromosome 1 paralogs are significant. ***p<0.001, *p<0.05, #p<0.1 (adjusted for false discovery rate). (TIF) [file pone.0096376.s001.tif]
