# Supplementary material for: Large Sex Differences in Chicken Behavior and Brain Gene Expression Coincide with Few Differences in Promoter DNA-Methylation
Source: PLoS One. 2014 Apr 29;9(4):e96376. doi: 10.1371/journal.pone.0096376 (PMC4004567; doi:10.1371/journal.pone.0096376)
Supplement: Table S1 — Significantly differentially expressed probesets in females related to males. (PDF) [file pone.0096376.s002.pdf]

Table S1. Significantly differentially expressed probesets in females related to males.

		Red Junglefowl (RJF)		*White Leghorn (WL)*			
Affymetrix ID	***Gene name***	***FC***	***p-value (FDR)***	***FC***	***p-value (FDR)***	***Best alignments***	***Type***
Gga.14796.1.S1_at	---	3.14	<0.0001	3.49	<0.0001	---	Sign. WL and RJF
GgaAffx.25782.2.S1_at	LOC427010	2.38	<0.0001	2.06	<0.0001	chr1:165954820-165965625	Sign. WL and RJF
GgaAffx.25782.2.S1_s_at	ZFR	1.09	<0.0001	0.98	<0.0001	chr1:165954820-165965625	Sign. WL and RJF
GgaAffx.25782.3.S1_s_at	LOC427010	0.92	<0.0001	0.95	<0.0001	chr1:165973581-165980409	Sign. WL and RJF
GgaAffx.5172.1.S1_at	LOC425409	1.01	<0.0001	1.06	<0.0001	chrUn_random:1118897-1129061	Sign. WL and RJF
GgaAffx.20721.1.S1_s_at	RCJMB04_17o18	-0.83	<0.0001	-0.79	<0.01	chrUn_random:15509070-15512734	Sign. WL and RJF
GgaAffx.8764.1.S1_s_at	RCJMB04_14b4	-0.81	<0.0001	-0.84	<0.001	chrUn_random:23438811-23440772	Sign. WL and RJF
GgaAffx.12386.1.S1_at	RCJMB04_14b4	-0.80	<0.001	-0.84	<0.001	chrUn_random:23438894-23441308	Sign. WL and RJF
Gga.11922.1.S1_at	BTF3	-0.64	<0.01	-0.64	<0.05	chrUn_random:31602735-31609298	Sign. WL and RJF
GgaAffx.12965.1.S1_at	ANKRA2	-0.82	<0.0001	-0.86	<0.001	chrUn_random:31620987-31628010	Sign. WL and RJF
GgaAffx.8604.1.S1_at	UTP15	-0.72	<0.001	-0.68	<0.05	chrUn_random:31628268-31638620	Sign. WL and RJF
GgaAffx.12834.1.S1_at	HNRPK	5.80	<0.0001	5.92	<0.0001	chrUn_random:38144124-38164861	Sign. WL and RJF
Gga.19575.1.S1_at	HNRPK	4.69	<0.0001	5.27	<0.0001	chrUn_random:38164764-38165589	Sign. WL and RJF
Gga.19575.1.A1_at	HNRPK	3.44	<0.0001	3.33	<0.0001	chrUn_random:38164764-38165589	Sign. WL and RJF
GgaAffx.11934.1.S1_s_at	DHFR	-0.63	<0.01	-0.63	<0.05	chrUn_random:40656761-40673403	Sign. WL and RJF
Gga.2883.1.S1_at	DHFR	-0.94	<0.0001	-0.78	<0.01	chrUn_random:40656761-40674166	Sign. WL and RJF
Gga.3078.1.S1_at	LOC430910	4.54	<0.0001	5.23	<0.0001	chrUn_random:47165755-47173919	Sign. WL and RJF
Gga.11630.2.S1_at	LOC426615	1.63	<0.0001	1.65	<0.0001	chrUn_random:53417660-53427017	Sign. WL and RJF
Gga.11630.2.S1_a_at	LOC426615	0.77	<0.001	0.70	<0.01	chrUn_random:53417660-53427017	Sign. WL and RJF
GgaAffx.25232.1.S1_s_at	GADD45B	-0.72	<0.001	-0.62	<0.05	chrUn_random:63869374-63870806	Sign. WL and RJF
GgaAffx.21598.1.S1_s_at	LOC425502	5.21	<0.0001	5.12	<0.0001	chrUn_random:8085899-8099453	Sign. WL and RJF
Gga.9791.1.S1_at	LOC427134	1.10	<0.0001	1.53	<0.0001	chrW:120571-127776	Sign. WL and RJF
Gga.15312.1.S1_a_at	UBAP2	1.49	<0.0001	1.96	<0.0001	chrW:72271-94265	Sign. WL and RJF
Gga.12737.1.S1_at	LOC426031	2.05	<0.0001	1.82	<0.0001	chrW_random:281584-292401	Sign. WL and RJF
Gga.14146.1.S1_at	LOC426031	0.92	<0.0001	0.92	<0.0001	chrW_random:292571-327719	Sign. WL and RJF
GgaAffx.20558.1.S1_at	CHD1	4.25	<0.0001	4.94	<0.0001	chrW_random:453596-454479	Sign. WL and RJF
Gga.8463.1.S1_s_at	CHD1	3.60	<0.0001	3.86	<0.0001	chrW_random:453596-454479	Sign. WL and RJF
Gga.7309.1.S1_at	LOC431003	2.29	<0.0001	2.39	<0.0001	chrW_random:468914-502728	Sign. WL and RJF
Gga.9713.2.S1_a_at	HINTW	8.51	<0.0001	8.49	<0.0001	chrW_random:581356-602643	Sign. WL and RJF
Gga.9338.1.S1_at	HINTW	2.03	<0.0001	1.42	<0.0001	chrW_random:591438-603267	Sign. WL and RJF
Gga.1845.1.S1_at	SPIN1	5.32	<0.0001	5.12	<0.0001	chrW_random:67979-154333	Sign. WL and RJF
Gga.16837.1.S1_at	NIPBL	0.81	<0.0001	0.88	<0.001	chrZ:10765084-10779413	Sign. WL and RJF
Gga.1946.1.S1_at	LOC427440	-0.89	<0.0001	-0.71	<0.01	chrZ:10832481-10846719	Sign. WL and RJF
GgaAffx.2307.1.S1_s_at	NUP155	-0.78	<0.001	-0.84	<0.001	chrZ:10903886-10935558	Sign. WL and RJF
GgaAffx.2320.1.S1_at	WDR70	-1.07	<0.0001	-0.95	<0.0001	chrZ:10937343-11080300	Sign. WL and RJF
Gga.16087.1.S1_at	LOC427191	-0.88	<0.0001	-0.83	<0.001	chrZ:12693736-12697319	Sign. WL and RJF
Gga.161.1.S1_at	SMAD2	-0.93	<0.0001	-0.81	<0.01	chrZ:1290955-1331604	Sign. WL and RJF
Gga.463.1.S1_at	MRPS30	-0.82	<0.0001	-0.93	<0.0001	chrZ:13609764-13870784	Sign. WL and RJF
Gga.9572.2.S1_s_at	MOCS2	-1.11	<0.0001	-1.02	<0.0001	chrZ:15250981-15256126	Sign. WL and RJF
Gga.12875.1.S1_at	HDHD2	-0.82	<0.0001	-0.99	<0.0001	chrZ:1534850-1541006	Sign. WL and RJF
Gga.3232.1.S1_at	NDUFS4	-1.00	<0.0001	-0.87	<0.001	chrZ:15447712-15474329	Sign. WL and RJF
GgaAffx.11436.1.S1_at	SKIV2L2	-0.98	<0.0001	-0.86	<0.001	chrZ:16102169-16145323	Sign. WL and RJF
Gga.9870.1.S1_s_at	SKIV2L2	-0.90	<0.0001	-0.88	<0.001	chrZ:16110096-16141691	Sign. WL and RJF
Gga.13230.1.S1_s_at	LOC770222	-0.61	<0.05	-0.76	<0.01	chrZ:16820497-16827908	Sign. WL and RJF
Gga.15137.1.S1_at	LOC769469	-0.74	<0.001	-0.69	<0.05	chrZ:17616045-17657651	Sign. WL and RJF
Gga.8219.1.S1_at	NDUFA12L	-0.60	<0.05	-0.70	<0.01	chrZ:18338289-18387063	Sign. WL and RJF
GgaAffx.9351.1.S1_at	LOC425720	0.97	<0.0001	0.88	<0.001	chrZ:18549817-18578697	Sign. WL and RJF
Gga.9720.1.S1_at	ATP5A1	-0.63	<0.01	-0.62	<0.05	chrZ:1940125-1946504	Sign. WL and RJF
GgaAffx.9364.1.S1_at	SFRS12IP1	-0.83	<0.0001	-0.88	<0.001	chrZ:19602295-19604473	Sign. WL and RJF
Gga.12941.1.S1_at	SDCCAG10	-0.78	<0.001	-0.62	<0.05	chrZ:19606909-19700941	Sign. WL and RJF
GgaAffx.9370.1.S1_at	PPWD1	-0.80	<0.001	-0.66	<0.05	chrZ:19982597-19996723	Sign. WL and RJF
GgaAffx.9374.1.S1_s_at	LOC427165	-0.75	<0.001	-0.73	<0.01	chrZ:20018729-20043194	Sign. WL and RJF
Gga.8469.1.S1_at	SLC30A5	-0.57	<0.05	-0.67	<0.05	chrZ:21302704-21303301	Sign. WL and RJF
Gga.9232.1.S1_at	MRPS36	-0.82	<0.0001	-0.80	<0.01	chrZ:21336850-21343473	Sign. WL and RJF
Gga.9685.1.S1_at	LOC771294	-0.64	<0.01	-0.77	<0.01	chrZ:21346549-21368036	Sign. WL and RJF
Gga.13994.1.S1_at	SCAMP1	-0.58	<0.05	-0.70	<0.01	chrZ:22156475-22192177	Sign. WL and RJF
Gga.5865.1.S1_at	TBCA	-1.12	<0.0001	-0.85	<0.001	chrZ:22531889-22564376	Sign. WL and RJF
Gga.12383.1.S1_at	CRHBP	-0.85	<0.0001	-0.64	<0.05	chrZ:22820329-22828750	Sign. WL and RJF
Gga.11441.1.S1_s_at	TINP1	-0.76	<0.001	-0.72	<0.01	chrZ:23675936-23679829	Sign. WL and RJF
GgaAffx.1097.1.S1_at	SETBP1	-0.63	<0.01	-0.63	<0.05	chrZ:2419077-2427279	Sign. WL and RJF
Gga.12029.1.S1_at	MRPS27	-0.72	<0.001	-0.83	<0.001	chrZ:26136678-26183146	Sign. WL and RJF
GgaAffx.12333.1.S1_s_at	RCJMB04_13c10	-0.58	<0.05	-0.63	<0.05	chrZ:26477352-26497485	Sign. WL and RJF
Gga.8237.1.S1_a_at	LOC770630	3.71	<0.0001	3.73	<0.0001	chrZ:27961979-27964669	Sign. WL and RJF
Gga.12454.1.S1_at	RLN3	1.72	<0.0001	1.52	<0.0001	chrZ:28156506-28159178	Sign. WL and RJF
Gga.19311.1.S1_s_at	ST8SIA3	-0.88	<0.0001	-0.86	<0.001	chrZ:317061-320461	Sign. WL and RJF
GgaAffx.21830.1.S1_at	ST8SIA3	-0.94	<0.0001	-0.79	<0.01	chrZ:317109-320470	Sign. WL and RJF
Gga.10398.1.S1_at	RIT2	-0.59	<0.05	-0.62	<0.05	chrZ:3240320-3301834	Sign. WL and RJF
GgaAffx.12114.1.S1_at	RCJMB04_9g9	-0.75	<0.001	-0.91	<0.001	chrZ:33363341-33382892	Sign. WL and RJF
GgaAffx.9584.1.S1_at	ADFP	-0.75	<0.001	-0.77	<0.01	chrZ:33391453-33395569	Sign. WL and RJF
Gga.4389.1.S1_a_at	RPS6	-0.55	<0.05	-0.64	<0.05	chrZ:33519934-33523885	Sign. WL and RJF
GgaAffx.9574.1.S1_s_at	KIAA1797	-0.75	<0.001	-0.76	<0.01	chrZ:33964854-34064295	Sign. WL and RJF
Gga.15983.1.S1_s_at	FXN	-0.90	<0.0001	-0.85	<0.001	chrZ:34238873-34249760	Sign. WL and RJF
GgaAffx.12402.1.S1_s_at	RCJMB04_14g12	-0.70	<0.01	-0.75	<0.01	chrZ:34629092-34685402	Sign. WL and RJF
GgaAffx.12473.1.S1_at	ZFAND5	-0.79	<0.001	-0.82	<0.001	chrZ:35431237-35446144	Sign. WL and RJF
GgaAffx.12473.1.S1_s_at	ZFAND5	-0.76	<0.001	-0.78	<0.01	chrZ:35431237-35446144	Sign. WL and RJF
Gga.4119.1.S1_at	ALDH1A1	-0.58	<0.05	-0.72	<0.01	chrZ:35596867-35649840	Sign. WL and RJF
Gga.19295.1.S1_at	LOC427259	-0.61	<0.05	-0.99	<0.0001	chrZ:36900804-36906630	Sign. WL and RJF
GgaAffx.9663.1.S1_at	CEP78	-0.72	<0.001	-0.77	<0.01	chrZ:37523068-37533365	Sign. WL and RJF
Gga.8770.2.S1_a_at	C9orf103	-0.73	<0.001	-0.81	<0.01	chrZ:39437468-39446503	Sign. WL and RJF
Gga.11444.1.S1_s_at	C9orf64	-0.77	<0.001	-0.85	<0.001	chrZ:39544138-39553136	Sign. WL and RJF
Gga.4811.1.S1_s_at	---	-0.63	<0.01	-0.68	<0.05	chrZ:39553279-39570110	Sign. WL and RJF
Gga.4811.2.S1_a_at	HNRNPK	-0.65	<0.01	-0.64	<0.05	chrZ:39553830-39562775	Sign. WL and RJF
GgaAffx.13129.1.S1_at	RMI1	-0.61	<0.05	-0.71	<0.01	chrZ:39572877-39576733	Sign. WL and RJF
GgaAffx.7995.2.S1_s_at	MAK10	-0.80	<0.001	-0.83	<0.001	chrZ:40230276-40255024	Sign. WL and RJF
Gga.12971.1.S1_at	LOC427469	-0.88	<0.0001	-0.79	<0.01	chrZ:41469381-41493202	Sign. WL and RJF
Gga.11727.1.S1_at	LOC427475	-0.92	<0.0001	-0.92	<0.001	chrZ:41503837-41523915	Sign. WL and RJF
Gga.11727.3.S1_a_at	LOC427475	-0.67	<0.01	-0.72	<0.01	chrZ:41513555-41513663	Sign. WL and RJF
Gga.4322.1.S1_at	SPIN1	-0.76	<0.001	-0.74	<0.01	chrZ:42620756-42648541	Sign. WL and RJF
Gga.9717.2.S1_s_at	SECISBP2	-0.59	<0.05	-0.67	<0.05	chrZ:42832607-42842833	Sign. WL and RJF
Gga.11726.1.S1_a_at	GADD45G	-0.79	<0.001	-0.79	<0.01	chrZ:43014620-43015472	Sign. WL and RJF
GgaAffx.24208.1.S1_at	SPTLC1	-0.80	<0.001	-0.74	<0.01	chrZ:43939897-43974064	Sign. WL and RJF
Gga.15824.1.S1_at	LYRM7	-0.85	<0.0001	-0.92	<0.0001	chrZ:44154455-44163095	Sign. WL and RJF

Gga.229.1.S1_at	HINT1	-0.67	<0.01	-0.75	<0.01	chrZ:44169862-44173887	Sign. WL and RJF
GgaAffx.21466.1.S1_s_at	YTHDC2	-0.90	<0.0001	-0.73	<0.01	chrZ:44852613-44856184	Sign. WL and RJF
GgaAffx.21466.1.S1_at	YTHDC2	-0.71	<0.01	-0.73	<0.01	chrZ:44852613-44856184	Sign. WL and RJF
Gga.9993.1.S1_at	SRP19	-0.81	<0.0001	-0.75	<0.01	chrZ:45167634-45174888	Sign. WL and RJF
GgaAffx.12320.1.S1_at	STARD4	-0.82	<0.0001	-0.79	<0.01	chrZ:45677855-45686316	Sign. WL and RJF
Gga.15911.1.S1_s_at	WDR36	-0.78	<0.001	-0.90	<0.001	chrZ:45899198-45907703	Sign. WL and RJF
GgaAffx.13113.1.S1_at	WDR36	-0.81	<0.0001	-0.79	<0.01	chrZ:45899205-45932568	Sign. WL and RJF
Gga.19662.1.S1_at	LOC415615	-1.18	<0.0001	-1.07	<0.0001	chrZ:46887905-46892854	Sign. WL and RJF
Gga.2083.1.S1_at	TXNL1	-0.87	<0.0001	-0.84	<0.001	chrZ:476762-491404	Sign. WL and RJF
GgaAffx.9704.1.S1_at	TMEM157	-0.78	<0.001	-0.95	<0.0001	chrZ:49789484-49803691	Sign. WL and RJF
GgaAffx.9739.1.S1_s_at	RIOK2	-0.60	<0.05	-0.70	<0.01	chrZ:50427859-50439732	Sign. WL and RJF
GgaAffx.12693.1.S1_at	RIOK2	-0.60	<0.05	-0.63	<0.05	chrZ:50427859-50439884	Sign. WL and RJF
Gga.4834.2.S1_at	RCJMB04_33p3	-0.91	<0.0001	-0.87	<0.001	chrZ:50949567-50964982	Sign. WL and RJF
GgaAffx.13170.1.S1_s_at	RCJMB04_33p3	-0.75	<0.001	-0.66	<0.05	chrZ:50949592-50965043	Sign. WL and RJF
Gga.4834.3.S1_at	RCJMB04_33p3	-0.84	<0.0001	-0.78	<0.01	chrZ:50964178-50965041	Sign. WL and RJF
GgaAffx.24658.1.S1_at	PIGG	-0.81	<0.0001	-0.97	<0.0001	chrZ:52129738-52212984	Sign. WL and RJF
Gga.15345.1.S1_s_at	TMEM175	-0.74	<0.001	-0.81	<0.01	chrZ:52357608-52364156	Sign. WL and RJF
Gga.8503.1.S1_at	NIPSNAP3A	-0.76	<0.001	-0.78	<0.01	chrZ:53588271-53597988	Sign. WL and RJF
Gga.11483.1.S1_at	FSD1L	-0.69	<0.01	-0.90	<0.001	chrZ:53895493-53920158	Sign. WL and RJF
GgaAffx.24485.1.S1_s_at	RCJMB04_11e18	-0.84	<0.0001	-0.66	<0.05	chrZ:55885794-55898342	Sign. WL and RJF
Gga.1140.1.S1_at	RCJMB04_11e18	-0.89	<0.0001	-0.90	<0.001	chrZ:55892268-55899679	Sign. WL and RJF
GgaAffx.12297.1.S1_s_at	ARSK	-1.12	<0.0001	-1.09	<0.0001	chrZ:56304344-56315471	Sign. WL and RJF
GgaAffx.9286.1.S1_at	KIAA0372	-0.74	<0.001	-0.75	<0.01	chrZ:56334524-56357555	Sign. WL and RJF
Gga.19460.1.S1_at	ANKRD32	-0.93	<0.0001	-0.98	<0.0001	chrZ:56699869-56707501	Sign. WL and RJF
GgaAffx.12343.1.S1_s_at	C5orf21	-0.77	<0.001	-0.83	<0.001	chrZ:56992246-57259077	Sign. WL and RJF
Gga.6910.1.A1_at	RCJMB04_38d18	-0.60	<0.05	-0.62	<0.05	chrZ:58551595-58552197	Sign. WL and RJF
Gga.9278.1.S1_at	CETN3	-0.89	<0.0001	-0.87	<0.001	chrZ:58564876-58581550	Sign. WL and RJF
Gga.9802.1.S1_at	CCNH	-0.83	<0.0001	-0.70	<0.01	chrZ:59652375-59662244	Sign. WL and RJF
Gga.11839.1.S1_s_at	CCNH	-0.92	<0.0001	-0.80	<0.01	chrZ:59652426-59661610	Sign. WL and RJF
Gga.14265.1.S1_at	RASA1	-0.78	<0.001	-0.75	<0.01	chrZ:59664414-59664606	Sign. WL and RJF
Gga.6171.2.S1_at	COX7C	-0.77	<0.001	-0.88	<0.001	chrZ:59900915-59904126	Sign. WL and RJF
GgaAffx.24733.1.S1_s_at	XRCC4	-0.70	<0.01	-0.67	<0.05	chrZ:61443250-61535260	Sign. WL and RJF
Gga.11793.1.S1_at	ATG10	-0.81	<0.0001	-0.75	<0.01	chrZ:62040471-62124175	Sign. WL and RJF
GgaAffx.9938.1.S1_at	LOC427318	-0.79	<0.001	-0.89	<0.001	chrZ:62614319-62667651	Sign. WL and RJF
GgaAffx.3134.1.S1_at	RCJMB04_1f9	-0.93	<0.0001	-0.68	<0.05	chrZ:62772410-62773050	Sign. WL and RJF
GgaAffx.11764.1.S1_at	RCJMB04_5a23	-0.79	<0.001	-0.72	<0.01	chrZ:63651117-63671625	Sign. WL and RJF
GgaAffx.9904.1.S1_s_at	RCJMB04_5a23	-0.77	<0.001	-0.68	<0.05	chrZ:63651457-63669020	Sign. WL and RJF
GgaAffx.11916.1.S1_s_at	MRPL50	-0.97	<0.0001	-0.95	<0.0001	chrZ:63710371-63711148	Sign. WL and RJF
GgaAffx.12705.1.S1_s_at	C9orf80	-0.84	<0.0001	-0.77	<0.01	chrZ:64463284-64474293	Sign. WL and RJF
Gga.2433.1.S1_at	---	-0.87	<0.0001	-0.70	<0.01	chrZ:64567064-64579646	Sign. WL and RJF
GgaAffx.25482.1.S1_at	C18orf10	-0.79	<0.001	-0.91	<0.001	chrZ:6624235-6647674	Sign. WL and RJF
GgaAffx.1170.1.S1_at	PLAA	-0.83	<0.0001	-0.75	<0.01	chrZ:66790625-66807684	Sign. WL and RJF
Gga.12675.1.S1_at	LOC427367	-0.62	<0.01	-0.79	<0.01	chrZ:66808017-66841141	Sign. WL and RJF
GgaAffx.12938.13.S1_at	UBE2R2	-0.58	<0.05	-0.64	<0.05	chrZ:6816941-6872776	Sign. WL and RJF
Gga.9488.1.S1_at	APTX	-0.70	<0.01	-0.84	<0.001	chrZ:68661014-68669891	Sign. WL and RJF
Gga.3310.1.S1_at	XPA	-0.76	<0.001	-0.85	<0.001	chrZ:68836469-68837258	Sign. WL and RJF
Gga.2614.1.A1_at	TMOD1	-0.62	<0.01	-0.86	<0.001	chrZ:68867405-68883975	Sign. WL and RJF
Gga.3546.1.S1_at	HSD17B4	-0.81	<0.0001	-0.80	<0.01	chrZ:69621720-69678124	Sign. WL and RJF
GgaAffx.12060.1.S1_at	COMMD10	-0.68	<0.01	-0.78	<0.01	chrZ:70838334-70934606	Sign. WL and RJF
GgaAffx.1544.1.S1_s_at	COMMD10	-0.79	<0.001	-0.71	<0.01	chrZ:70838829-70934394	Sign. WL and RJF
Gga.9200.1.S1_s_at	AP3S1	-0.58	<0.05	-0.71	<0.01	chrZ:71017173-71044186	Sign. WL and RJF
Gga.12541.1.S1_at	NUDT2	-0.90	<0.0001	-0.97	<0.0001	chrZ:7103206-7109726	Sign. WL and RJF
GgaAffx.13180.1.S1_s_at	MTAP	-0.79	<0.001	-0.85	<0.001	chrZ:71968345-71996016	Sign. WL and RJF
Gga.19885.1.S1_at	CCDC112	-0.78	<0.001	-0.99	<0.0001	chrZ:72191743-72201153	Sign. WL and RJF
Gga.11454.1.S1_at	ALDH7A1	-0.81	<0.0001	-0.85	<0.001	chrZ:72301375-72320595	Sign. WL and RJF
Gga.10539.2.S1_a_at	SRFBP1	-0.68	<0.01	-1.01	<0.0001	chrZ:73534307-73593876	Sign. WL and RJF
Gga.11468.1.S1_a_at	PPIC	-0.73	<0.001	-0.62	<0.05	chrZ:73945599-73950771	Sign. WL and RJF
GgaAffx.12368.1.S1_s_at	DCTN3	-0.91	<0.0001	-0.92	<0.001	chrZ:7400105-7404679	Sign. WL and RJF
Gga.8188.1.S1_a_at	---	-0.77	<0.001	-0.90	<0.001	chrZ:74124445-74125003	Sign. WL and RJF
Gga.11290.1.S1_at	LOC768709	-0.87	<0.0001	-0.77	<0.01	chrZ:74169618-74175695	Sign. WL and RJF
GgaAffx.26489.1.S1_at	POLR1E	-0.82	<0.0001	-0.73	<0.01	chrZ:74199055-74203036	Sign. WL and RJF
Gga.7241.1.S1_at	GRHPR	-0.82	<0.0001	-0.95	<0.0001	chrZ:74230601-74235890	Sign. WL and RJF
Gga.13352.1.S1_at	ZCCHC7	-0.55	<0.05	-0.61	<0.05	chrZ:74251024-74252249	Sign. WL and RJF
GgaAffx.11388.1.S1_at	VCP	-0.66	<0.01	-0.64	<0.05	chrZ:7931928-7954971	Sign. WL and RJF
GgaAffx.1592.2.S1_at	KIAA0258	-0.72	<0.001	-0.73	<0.01	chrZ:8455682-8462339	Sign. WL and RJF
Gga.3315.1.S1_a_at	MRPL17	-0.96	<0.0001	-0.77	<0.01	chrZ:8638818-8639846	Sign. WL and RJF
Gga.3315.2.S1_a_at	MRPL17	-1.09	<0.0001	-0.89	<0.001	chrZ:8638820-8639809	Sign. WL and RJF
Gga.3315.2.S1_x_at	MRPL17	-0.99	<0.0001	-0.78	<0.01	chrZ:8638820-8639809	Sign. WL and RJF
GgaAffx.11719.1.S1_s_at	ACAA2	-0.73	<0.001	-0.68	<0.05	chrZ:864203-877738	Sign. WL and RJF
GgaAffx.11726.1.S1_at	RCJMB04_4k14	-0.81	<0.0001	-0.77	<0.01	chrZ:9466481-9482354	Sign. WL and RJF
GgaAffx.2056.1.S1_s_at	RCJMB04_4k14	-0.84	<0.0001	-0.87	<0.001	chrZ:9466884-9482067	Sign. WL and RJF
Gga.12669.1.S1_at	RAD1	-0.89	<0.0001	-0.85	<0.001	chrZ:9913430-9917002	Sign. WL and RJF
GgaAffx.11728.1.S1_s_at	BXDC2	-0.93	<0.0001	-0.76	<0.01	chrZ:9918981-9922558	Sign. WL and RJF
Gga.17540.1.S1_at	ACOT9	NS	NS	-0.66	<0.05	chr1:121798476-121800274	Sign. WL
GgaAffx.8168.1.S1_s_at	SLC13A4	NS	NS	0.84	<0.001	chr1:60454429-60515119	Sign. WL
Gga.11808.1.S1_at	PYROXD1	NS	NS	0.62	<0.05	chr1:67455630-67470742	Sign. WL
GgaAffx.3400.1.S1_s_at	C16orf73	NS	NS	0.62	<0.05	chr14:6082271-6088607	Sign. WL
Gga.7378.1.S1_at	PDE6G	NS	NS	1.60	<0.0001	chr18:9170776-9171418	Sign. WL
Gga.17147.1.S1_s_at	CCDC127	NS	NS	0.77	<0.01	chr2:87985511-87987029	Sign. WL
Gga.6201.1.S1_at	ISG12-2	NS	NS	-0.71	<0.01	chr2:92043529-92046970	Sign. WL
GgaAffx.4201.1.S1_s_at	USP2	NS	NS	0.62	<0.05	chr24:4214460-4220496	Sign. WL
Gga.3296.1.S1_at	GTSF1	NS	NS	0.62	<0.05	chr27:1011614-1025910	Sign. WL
Gga.1622.1.S1_at	MANEA	NS	NS	0.61	<0.05	chr3:75857853-75858627	Sign. WL
Gga.15984.1.S1_at	CCNG2	NS	NS	0.71	<0.01	chr4:36165726-36169902	Sign. WL
Gga.3551.1.S1_at	SPP1	NS	NS	1.18	<0.0001	chr4:47107503-47110559	Sign. WL
Gga.1128.2.S1_a_at	SOD3	NS	NS	0.64	<0.05	chr4:76179385-76181731	Sign. WL
Gga.552.1.S1_at	DIO3	NS	NS	0.74	<0.01	chr5:51792817-51794155	Sign. WL
GgaAffx.5665.1.S1_at	VWA2	NS	NS	-0.68	<0.05	chr6:29197628-29210779	Sign. WL
Gga.11095.1.S1_s_at	SPOPL	NS	NS	0.64	<0.05	chr7:32994083-33017751	Sign. WL
GgaAffx.9275.1.S1_at	CHD1	NS	NS	0.71	<0.01	chrW_random:437861-450878	Sign. WL
GgaAffx.24537.1.S1_at	PARP8	NS	NS	-0.63	<0.05	chrZ:14212894-14321609	Sign. WL
Gga.8226.1.S1_s_at	LOC431580	NS	NS	-0.63	<0.05	chrZ:18915340-18949763	Sign. WL
Gga.7955.1.S1_at	SLC14A2	NS	NS	-0.62	<0.05	chrZ:2042236-2048956	Sign. WL
Gga.10791.1.S1_s_at	COL4A3BP	NS	NS	-0.73	<0.01	chrZ:23387542-23442430	Sign. WL
Gga.15064.1.S1_at	TNPO1	NS	NS	-0.68	<0.05	chrZ:25932295-25955137	Sign. WL
GgaAffx.9593.1.S1_at	C9orf39	NS	NS	-0.70	<0.01	chrZ:32677918-32685039	Sign. WL
Gga.18888.1.A1_at	MAK10	NS	NS	-0.63	<0.05	chrZ:40252844-40255288	Sign. WL

Gga.17317.1.S1_s_at	TRPV6	NS	NS	-0.74	<0.01	chrZ:41094904-41101596	Sign. WL
Gga.12503.2.S1_at	NXNL2	NS	NS	-0.86	<0.001	chrZ:42662496-42663156	Sign. WL
GgaAffx.11649.1.S1_at	RCJMB04_3e14	NS	NS	-0.63	<0.05	chrZ:43555227-43588754	Sign. WL
Gga.15316.1.S1_s_at	STARD4	NS	NS	-0.80	<0.01	chrZ:45682743-45683859	Sign. WL
Gga.832.1.S1_at	LOC396454	NS	NS	2.65	<0.0001	chrZ:52408698-52414029	Sign. WL
GgaAffx.9832.1.S1_at	FKTN	NS	NS	-0.78	<0.01	chrZ:53929929-53942504	Sign. WL
Gga.2141.2.S1_at	C5orf21	NS	NS	-0.90	<0.001	chrZ:57130372-57173562	Sign. WL
GgaAffx.25289.1.S1_at	RASA1	NS	NS	-0.61	<0.05	chrZ:59665193-59710880	Sign. WL
Gga.17469.1.S1_s_at	RAD17	NS	NS	-0.73	<0.01	chrZ:62774060-62790439	Sign. WL
Gga.4066.1.S1_at	SMC2	NS	NS	-0.64	<0.05	chrZ:65014193-65036340	Sign. WL
GgaAffx.1613.1.S1_at	RCJMB04_5p1	NS	NS	-0.63	<0.05	chrZ:6595264-6620306	Sign. WL
Gga.1495.2.S1_s_at	SNCAIP	NS	NS	-0.73	<0.01	chrZ:73745304-73770300	Sign. WL
Gga.10995.1.S1_s_at	C9orf23	NS	NS	-0.69	<0.05	chrZ:7394414-7397286	Sign. WL
GgaAffx.11974.1.S1_at	RCJMB04_7i6	NS	NS	-0.63	<0.05	chrZ:9721344-9743363	Sign. WL
Gga.10904.1.S1_at	AGXT2	NS	NS	-0.63	<0.05	chrZ:9938102-9940088	Sign. WL
Gga.19447.2.S1_s_at	LOC770705	-0.70	<0.01	NS	NS	---	Sign. RJF
Gga.4975.5.S1_a_at	TPM3	-0.64	<0.01	NS	NS	---	Sign. RJF
Gga.4975.5.S1_x_at	TPM3	-0.64	<0.01	NS	NS	---	Sign. RJF
GgaAffx.10196.3.S1_s_at	ITSN1	-0.72	<0.001	NS	NS	chr1:109022133-109028175	Sign. RJF
Gga.2896.1.S1_at	CBR1	-0.53	<0.05	NS	NS	chr1:110031351-110035333	Sign. RJF
Gga.11350.2.S1_at	ACE2	0.62	<0.01	NS	NS	chr1:125385168-125386739	Sign. RJF
GgaAffx.5027.1.S1_at	COG5	0.53	<0.05	NS	NS	chr1:15708716-15731315	Sign. RJF
Gga.19912.1.S1_at	LOC427010	0.53	<0.05	NS	NS	chr1:165971147-165977798	Sign. RJF
GgaAffx.25188.1.S1_at	KBTBD3	0.53	<0.05	NS	NS	chr1:185441837-185446240	Sign. RJF
Gga.9760.1.S1_at	C12orf5	0.61	<0.05	NS	NS	chr1:75468455-75488310	Sign. RJF
Gga.3807.1.S2_s_at	ALDH1A3	0.78	<0.001	NS	NS	chr10:19570925-19612702	Sign. RJF
GgaAffx.11898.1.S1_at	FBXO22	0.65	<0.01	NS	NS	chr10:4433990-4440640	Sign. RJF
Gga.15212.1.S1_s_at	CNTN3	-0.55	<0.05	NS	NS	chr12:17510323-17523754	Sign. RJF
Gga.6976.1.S1_at	LOC416235	0.54	<0.05	NS	NS	chr13:10614290-10624240	Sign. RJF
GgaAffx.9243.3.S1_s_at	CNOT6	0.63	<0.01	NS	NS	chr13:13986720-13996913	Sign. RJF
Gga.876.1.S1_s_at	B-G	0.66	<0.01	NS	NS	chr16:221974-224822	Sign. RJF
GgaAffx.2719.1.S1_s_at	LOC771897	0.58	<0.05	NS	NS	chr19:6268987-6273094	Sign. RJF
Gga.13339.1.S1_at	ST8SIA6	0.55	<0.05	NS	NS	chr2:19666197-19704978	Sign. RJF
GgaAffx.5986.1.S1_at	CALCR	0.56	<0.05	NS	NS	chr2:23010363-23064882	Sign. RJF
GgaAffx.3437.3.S1_at	PTH1R	-0.60	<0.05	NS	NS	chr2:3543596-3543792	Sign. RJF
GgaAffx.8120.2.S1_at	C6orf85	0.71	<0.01	NS	NS	chr2:67380636-67387653	Sign. RJF
Gga.5973.1.S1_at	APCDD1L	-0.59	<0.05	NS	NS	chr20:11113825-11128911	Sign. RJF
Gga.3242.2.S1_at	LOC769185	-1.17	<0.0001	NS	NS	chr20:12367161-12395342	Sign. RJF
GgaAffx.24375.2.S1_at	PLA2G5	0.54	<0.05	NS	NS	chr21:4893242-4900568	Sign. RJF
GgaAffx.25795.1.S1_s_at	COMP	-0.57	<0.05	NS	NS	chr28:2889937-2904753	Sign. RJF
GgaAffx.20768.1.S1_at	SLC4A1AP	1.32	<0.0001	NS	NS	chr3:28994967-29001132	Sign. RJF
Gga.8161.1.S1_s_at	C1orf57	0.54	<0.05	NS	NS	chr3:40764729-40778491	Sign. RJF
GgaAffx.11872.1.S1_at	C1orf57	0.67	<0.01	NS	NS	chr3:40764732-40777843	Sign. RJF
Gga.17069.1.S1_s_at	SERTAD2	0.53	<0.05	NS	NS	chr3:9730898-9742633	Sign. RJF
GgaAffx.12171.1.S1_s_at	RCJMB04_10b24	0.59	<0.05	NS	NS	chr3:98813588-98904285	Sign. RJF
GgaAffx.12585.1.S1_at	RCJMB04_18k24	0.60	<0.05	NS	NS	chr4:35021822-35040706	Sign. RJF
Gga.1849.1.S1_at	CHAT	0.55	<0.05	NS	NS	chr6:3933063-3964302	Sign. RJF
GgaAffx.5692.4.S1_s_at	RCJMB04_12b15	0.54	<0.05	NS	NS	chr7:15894934-15902562	Sign. RJF
Gga.13607.1.S1_at	---	0.54	<0.05	NS	NS	chrUn_random:52794596-52795891	Sign. RJF
Gga.19177.2.S1_at	LOC425502	0.84	<0.0001	NS	NS	chrUn_random:8079297-8091122	Sign. RJF
Gga.12002.1.S1_a_at	CAPSL	-0.69	<0.01	NS	NS	chrZ:10254114-10262495	Sign. RJF
Gga.13223.1.S1_at	TTC33	-0.61	<0.05	NS	NS	chrZ:12268599-12304231	Sign. RJF
Gga.3828.1.S1_at	EMB	-0.60	<0.05	NS	NS	chrZ:14147213-14166737	Sign. RJF
GgaAffx.24537.1.S1_s_at	PARP8	-0.80	<0.001	NS	NS	chrZ:14212894-14321609	Sign. RJF
Gga.9708.2.S1_a_at	PPAP2A	-0.69	<0.01	NS	NS	chrZ:16145633-16202871	Sign. RJF
GgaAffx.9335.1.S1_at	SLC38A9	-0.58	<0.05	NS	NS	chrZ:16229788-16265216	Sign. RJF
GgaAffx.9344.1.S1_s_at	GPBP1	-0.67	<0.01	NS	NS	chrZ:16894595-16913564	Sign. RJF
Gga.10660.2.S1_at	PLK2	-0.57	<0.05	NS	NS	chrZ:17389450-17395404	Sign. RJF
GgaAffx.24493.1.S1_at	PLK2	-0.61	<0.05	NS	NS	chrZ:17390040-17394824	Sign. RJF
Gga.11058.1.S1_at	ERCC8	-0.56	<0.05	NS	NS	chrZ:18298124-18338212	Sign. RJF
Gga.17588.1.S1_at	DIMT1L	-0.57	<0.05	NS	NS	chrZ:18865318-18870752	Sign. RJF
Gga.8226.1.S1_at	LOC431580	-0.70	<0.01	NS	NS	chrZ:18915340-18949763	Sign. RJF
GgaAffx.20659.1.S1_at	LOC769679	-0.60	<0.05	NS	NS	chrZ:18935601-18936564	Sign. RJF
GgaAffx.12568.1.S1_at	CCDC5	-0.71	<0.01	NS	NS	chrZ:1925178-1934125	Sign. RJF
GgaAffx.1153.1.S1_s_at	CCDC5	-0.63	<0.01	NS	NS	chrZ:1928270-1932660	Sign. RJF
GgaAffx.9361.1.S1_at	RNF180	-0.59	<0.05	NS	NS	chrZ:19448901-19520028	Sign. RJF
Gga.12212.1.S1_a_at	CENPK	-0.64	<0.01	NS	NS	chrZ:19959937-19982485	Sign. RJF
GgaAffx.11883.1.S1_at	SLC30A5	-0.64	<0.01	NS	NS	chrZ:21302704-21323826	Sign. RJF
Gga.944.1.S1_at	CENPH	-0.67	<0.01	NS	NS	chrZ:21329350-21335530	Sign. RJF
Gga.19614.1.S1_at	PAPD4	-0.54	<0.05	NS	NS	chrZ:21599064-21614031	Sign. RJF
GgaAffx.2751.1.S1_at	ARSB	-0.64	<0.01	NS	NS	chrZ:21997832-21998718	Sign. RJF
Gga.1233.2.S1_a_at	AP3B1	-0.55	<0.05	NS	NS	chrZ:22356768-22387932	Sign. RJF
GgaAffx.9504.1.S1_at	C5orf37	-0.63	<0.01	NS	NS	chrZ:23312271-23322955	Sign. RJF
GgaAffx.24549.1.S1_at	ANKRD31	-0.59	<0.05	NS	NS	chrZ:23527763-23552212	Sign. RJF
GgaAffx.9494.1.S1_at	LOC431595	-0.58	<0.05	NS	NS	chrZ:23651377-23669383	Sign. RJF
GgaAffx.9489.1.S1_s_at	GFM2	-0.60	<0.05	NS	NS	chrZ:23680812-23697691	Sign. RJF
Gga.10677.1.S1_a_at	PTCD2	-0.69	<0.01	NS	NS	chrZ:26119800-26136603	Sign. RJF
GgaAffx.6384.2.S1_s_at	RCJMB04_13c10	-0.60	<0.05	NS	NS	chrZ:26477833-26497452	Sign. RJF
GgaAffx.8117.1.S1_at	DMRT3	0.54	<0.05	NS	NS	chrZ:26801249-26801668	Sign. RJF
GgaAffx.24608.1.S1_at	C9orf93	-0.56	<0.05	NS	NS	chrZ:31720785-31866352	Sign. RJF
GgaAffx.25275.1.S1_at	RIT2	-0.92	<0.0001	NS	NS	chrZ:3301761-3323125	Sign. RJF
Gga.14002.2.S1_at	DENND4C	-0.85	<0.0001	NS	NS	chrZ:33439594-33462784	Sign. RJF
GgaAffx.21323.1.S1_at	KIAA1797	-0.75	<0.001	NS	NS	chrZ:33985322-34009788	Sign. RJF
Gga.6358.1.S1_at	C9orf61	-0.63	<0.01	NS	NS	chrZ:34366203-34368642	Sign. RJF
GgaAffx.24618.1.S1_s_at	ZFAND5	-0.60	<0.05	NS	NS	chrZ:35432721-35442607	Sign. RJF
Gga.2654.1.S1_at	C9orf95	-0.58	<0.05	NS	NS	chrZ:36391099-36397679	Sign. RJF
Gga.681.2.S1_a_at	NTRK2	-0.64	<0.01	NS	NS	chrZ:39776254-39872350	Sign. RJF
Gga.9179.1.S1_at	RCJMB04_1b10	-0.54	<0.05	NS	NS	chrZ:40797583-40839437	Sign. RJF
GgaAffx.23948.1.S1_at	LOC427469	-0.68	<0.01	NS	NS	chrZ:41491447-41498239	Sign. RJF
Gga.2855.1.S1_at	HABP4	-0.81	<0.0001	NS	NS	chrZ:41679664-41701846	Sign. RJF
Gga.11912.1.S1_at	DIRAS2	-0.57	<0.05	NS	NS	chrZ:43162132-43280348	Sign. RJF
GgaAffx.25327.1.S1_at	APC	-0.67	<0.01	NS	NS	chrZ:45196860-45274857	Sign. RJF
Gga.10382.1.S1_s_at	APC	-0.82	<0.0001	NS	NS	chrZ:45214288-45274849	Sign. RJF
Gga.2184.1.S1_at	C5orf13	-0.56	<0.05	NS	NS	chrZ:45605112-45625271	Sign. RJF
Gga.17282.1.S1_at	PAM	-0.59	<0.05	NS	NS	chrZ:48974989-49032577	Sign. RJF
Gga.4301.1.S1_at	CHD1	-0.56	<0.05	NS	NS	chrZ:50154415-50204555	Sign. RJF
Gga.4301.2.S1_a_at	CHD1	-0.54	<0.05	NS	NS	chrZ:50181097-50199702	Sign. RJF

Gga.7694.1.S1_at	MFSD7	-0.56	<0.05	NS	NS	chrZ:51629740-51632132	Sign. RJF
Gga.6091.1.S1_s_at	ATP5I	-0.57	<0.05	NS	NS	chrZ:52365085-52366954	Sign. RJF
Gga.10132.1.S1_at	RNF170	-0.54	<0.05	NS	NS	chrZ:52556997-52570420	Sign. RJF
Gga.8587.1.S1_at	LOC427125	-0.72	<0.001	NS	NS	chrZ:55189439-55200086	Sign. RJF
Gga.635.1.S1_at	GLRX	-0.59	<0.05	NS	NS	chrZ:56240542-56249419	Sign. RJF
GgaAffx.25682.1.S1_s_at	NEDD4L	-0.66	<0.01	NS	NS	chrZ:590978-629918	Sign. RJF
Gga.6171.1.S1_a_at	COX7C	-0.61	<0.05	NS	NS	chrZ:59900906-59904132	Sign. RJF
Gga.4974.1.S1_at	VCAN	-0.56	<0.05	NS	NS	chrZ:61307546-61406145	Sign. RJF
GgaAffx.24733.1.S1_at	XRCC4	-0.73	<0.001	NS	NS	chrZ:61443250-61535260	Sign. RJF
Gga.3441.2.S1_s_at	TMEM167	-0.59	<0.05	NS	NS	chrZ:61618461-61640282	Sign. RJF
Gga.13124.1.S1_at	LOC427324	-0.65	<0.01	NS	NS	chrZ:62003926-62019699	Sign. RJF
Gga.7337.1.S1_at	SSBP2	-0.55	<0.05	NS	NS	chrZ:62191512-62361161	Sign. RJF
Gga.2944.2.S1_a_at	NEDD4L	-0.54	<0.05	NS	NS	chrZ:626577-630210	Sign. RJF
GgaAffx.24720.1.S1_at	LOC768383	-0.60	<0.05	NS	NS	chrZ:62736226-62756460	Sign. RJF
GgaAffx.9996.2.S1_at	SUSD1	-0.57	<0.05	NS	NS	chrZ:64707508-64722460	Sign. RJF
Gga.18415.1.A1_at	ELAVL2	-0.55	<0.05	NS	NS	chrZ:65978273-65979579	Sign. RJF
Gga.1774.1.S1_at	C9orf82	-0.54	<0.05	NS	NS	chrZ:66771681-66788733	Sign. RJF
GgaAffx.25442.1.S1_s_at	SEMA6A	-0.71	<0.01	NS	NS	chrZ:70721111-70780614	Sign. RJF
Gga.11356.1.S1_s_at	ATG12	-0.68	<0.01	NS	NS	chrZ:71047297-71049202	Sign. RJF
Gga.7932.1.S1_s_at	ATG12	-0.71	<0.01	NS	NS	chrZ:71047356-71049202	Sign. RJF
Gga.16647.1.S1_at	DNAI1	-0.79	<0.001	NS	NS	chrZ:7336093-7390055	Sign. RJF
GgaAffx.3333.1.S1_at	SNCAIP	-0.78	<0.001	NS	NS	chrZ:73727423-73776692	Sign. RJF
Gga.10995.1.S1_at	C9orf23	-0.75	<0.001	NS	NS	chrZ:7394414-7397286	Sign. RJF
Gga.9925.1.S1_at	C9orf23	-0.65	<0.01	NS	NS	chrZ:7394417-7396762	Sign. RJF
Gga.3857.1.S1_s_at	FANCG	-0.61	<0.05	NS	NS	chrZ:7956394-7961602	Sign. RJF
GgaAffx.1592.1.S1_s_at	KIAA0258	-0.65	<0.01	NS	NS	chrZ:8455733-8462336	Sign. RJF
Gga.4975.2.S1_a_at	TPM3	-0.65	<0.01	NS	NS	chrZ:8546811-8554861	Sign. RJF
Gga.12332.1.S1_at	GOLPH3	-0.57	<0.05	NS	NS	chrZ:9011769-9044681	Sign. RJF
Gga.4786.1.S1_at	C18orf32	-0.66	<0.01	NS	NS	chrZ:968727-972168	Sign. RJF

Genebuild: WASHUC2.
