# Supplementary material for: Large Sex Differences in Chicken Behavior and Brain Gene Expression Coincide with Few Differences in Promoter DNA-Methylation
Source: PLoS One. 2014 Apr 29;9(4):e96376. doi: 10.1371/journal.pone.0096376 (PMC4004567; doi:10.1371/journal.pone.0096376)
Supplement: Table S2 — Significantly differentially methylated probes in females related to males. (PDF) [file pone.0096376.s003.pdf]

Table S2. Significantly differentially methylated probes in females related to males.

			Red Junglefowl		White Leghorn			
Spot ID	Probe start (bp)	Promoter range (bp)	Log2 FC	p-value (FDR)	Log2 FC	p-value (FDR)	Closest gene(s)	Type
CHR01FS165984037	165984037	chr1:165977159-165987659	2.38	0.016	1.81	0.018	ZFR	Sign. RJF and WL
CHRUNRFS022700108	22700108	chrUN_random:22699192-22709692	2.88	0.010	3.09	0.000	LOC429831; ZNF687	Sign. RJF and WL
CHRUNRFS022700255	22700255	chrUN_random:22699192-22709692	2.99	0.021	2.66	0.001	LOC429831; ZNF687	Sign. RJF and WL
CHRUNRFS022700359	22700359	chrUN_random:22699192-22709692	2.20	0.042	2.56	0.001	LOC429831; ZNF687	Sign. RJF and WL
CHRUNRFS022699719	22699719	chrUN_random:22699192-22709692	2.37	0.018	2.60	0.002	LOC429831; ZNF687	Sign. RJF and WL
CHRUNRFS022699894	22699894	chrUN_random:22699192-22709692	2.41	0.016	2.16	0.004	LOC429831; ZNF687	Sign. RJF and WL
CHRUNRFS047164881	47164881	chrUN_random:47158633-47169133	2.95	0.018	2.95	0.000	LOC430910; BTF3	Sign. RJF and WL
CHRUNRFS047164971	47164971	chrUN_random:47158633-47169133	2.52	0.008	2.71	0.001	LOC430910; BTF3	Sign. RJF and WL
CHRUNRFS047163667	47163667	chrUN_random:47158633-47169133	2.38	0.018	2.41	0.002	LOC430910; BTF3	Sign. RJF and WL
CHRZFS027968870	27968870	chrZ:27967699-27978199	-4.12	0.002	-3.49	0.000	Novel gene in MHM	Sign. RJF and WL
CHRZFS027971096	27971096	chrZ:27967699-27978199	-4.37	0.002	-3.53	0.000	Novel gene in MHM	Sign. RJF and WL
CHRZFS027968782	27968782	chrZ:27967699-27978199	-4.40	0.006	-3.59	0.000	Novel gene in MHM	Sign. RJF and WL
CHRZFS027970826	27970826	chrZ:27967699-27978199	-4.52	0.002	-3.48	0.000	Novel gene in MHM	Sign. RJF and WL
CHRZFS027970734	27970734	chrZ:27967699-27978199	-3.79	0.002	-3.44	0.000	Novel gene in MHM	Sign. RJF and WL
CHRZFS027971438	27971438	chrZ:27967699-27978199	-4.01	0.006	-3.24	0.000	Novel gene in MHM	Sign. RJF and WL
CHRZFS027971518	27971518	chrZ:27967699-27978199	-3.28	0.034	-3.56	0.000	Novel gene in MHM	Sign. RJF and WL
CHRZFS027971708	27971708	chrZ:27967699-27978199	-3.59	0.006	-3.20	0.000	Novel gene in MHM	Sign. RJF and WL
CHRZFS027970474	27970474	chrZ:27967699-27978199	-3.52	0.002	-3.26	0.000	Novel gene in MHM	Sign. RJF and WL
CHRZFS027968948	27968948	chrZ:27967699-27978199	-4.03	0.002	-3.19	0.000	Novel gene in MHM	Sign. RJF and WL
CHRZFS027971864	27971864	chrZ:27967699-27978199	-3.58	0.002	-3.02	0.000	Novel gene in MHM	Sign. RJF and WL
CHRZFS027968698	27968698	chrZ:27967699-27978199	-4.08	0.016	-3.01	0.000	Novel gene in MHM	Sign. RJF and WL
CHRZFS027969122	27969122	chrZ:27967699-27978199	-2.91	0.013	-2.88	0.000	Novel gene in MHM	Sign. RJF and WL
CHRZFS027971784	27971784	chrZ:27967699-27978199	-3.32	0.003	-3.14	0.000	Novel gene in MHM	Sign. RJF and WL
CHRZFS027970994	27970994	chrZ:27967699-27978199	-3.57	0.002	-3.27	0.000	Novel gene in MHM	Sign. RJF and WL
CHRZFS027970914	27970914	chrZ:27967699-27978199	-3.42	0.003	-3.09	0.000	Novel gene in MHM	Sign. RJF and WL
CHRZFS027969820	27969820	chrZ:27967699-27978199	-3.18	0.006	-3.18	0.000	Novel gene in MHM	Sign. RJF and WL
CHRZFS027970404	27970404	chrZ:27967699-27978199	-3.02	0.006	-2.77	0.000	Novel gene in MHM	Sign. RJF and WL
CHRZFS027971272	27971272	chrZ:27967699-27978199	-4.41	0.007	-2.76	0.000	Novel gene in MHM	Sign. RJF and WL
CHRZFS027971170	27971170	chrZ:27967699-27978199	-3.53	0.046	-2.73	0.000	Novel gene in MHM	Sign. RJF and WL
CHRZFS027969480	27969480	chrZ:27967699-27978199	-2.99	0.006	-2.67	0.001	Novel gene in MHM	Sign. RJF and WL
CHRZFS027969046	27969046	chrZ:27967699-27978199	-3.33	0.002	-2.94	0.001	Novel gene in MHM	Sign. RJF and WL
CHRZFS027970316	27970316	chrZ:27967699-27978199	-2.87	0.005	-2.72	0.001	Novel gene in MHM	Sign. RJF and WL
CHRZFS027971618	27971618	chrZ:27967699-27978199	-3.26	0.006	-2.82	0.001	Novel gene in MHM	Sign. RJF and WL
CHRZFS027970570	27970570	chrZ:27967699-27978199	-3.87	0.002	-3.15	0.001	Novel gene in MHM	Sign. RJF and WL
CHRZFS027969390	27969390	chrZ:27967699-27978199	-2.74	0.006	-2.67	0.001	Novel gene in MHM	Sign. RJF and WL
CHRZFS027969638	27969638	chrZ:27967699-27978199	-3.05	0.003	-2.58	0.001	Novel gene in MHM	Sign. RJF and WL
CHRZFS027969206	27969206	chrZ:27967699-27978199	-2.90	0.006	-2.84	0.002	Novel gene in MHM	Sign. RJF and WL
CHRZFS027969904	27969904	chrZ:27967699-27978199	-2.76	0.007	-2.61	0.002	Novel gene in MHM	Sign. RJF and WL
CHRZFS027972400	27972400	chrZ:27967699-27978199	-3.42	0.002	-2.96	0.002	Novel gene in MHM	Sign. RJF and WL
CHRZFS027972058	27972058	chrZ:27967699-27978199	-2.39	0.014	-2.14	0.004	Novel gene in MHM	Sign. RJF and WL
CHRZFS027970210	27970210	chrZ:27967699-27978199	-2.67	0.016	-2.39	0.007	Novel gene in MHM	Sign. RJF and WL
CHRZFS027971964	27971964	chrZ:27967699-27978199	-2.37	0.021	-2.06	0.010	Novel gene in MHM	Sign. RJF and WL
CHRZFS027970660	27970660	chrZ:27967699-27978199	-2.48	0.014	-1.98	0.012	Novel gene in MHM	Sign. RJF and WL
CHRZFS027969724	27969724	chrZ:27967699-27978199	-2.76	0.018	-1.88	0.014	Novel gene in MHM	Sign. RJF and WL
CHRZFS027972304	27972304	chrZ:27967699-27978199	-2.07	0.047	-2.06	0.016	Novel gene in MHM	Sign. RJF and WL
CHRZFS027972144	27972144	chrZ:27967699-27978199	-2.40	0.016	-1.91	0.034	Novel gene in MHM	Sign. RJF and WL
CHR01FS165983959	165983959	chr1:165977159-165987659	2.04	0.035	NA	NA	ZFR	Sign. RJF
CHR01FS067458774	67458774	chr1:67448444-67458944	-2.64	0.016	NA	NA	PYROXD1	Sign. RJF
CHR04FS068950313	68950313	chr4:68945836-68956336	3.44	0.013	NA	NA	GABRA2	Sign. RJF
CHR04FS087527256	87527256	chr4:87522175-87532675	-2.50	0.024	NA	NA	LOC776823; LOC777427; LOC	Sign. RJF
CHR05FS015215576	15215576	chr5:15209550-15229085	-2.09	0.042	NA	NA	LOC770392; CTSD	Sign. RJF
CHR06FS031517001	31517001	chr6:31506915-31517415	-3.59	0.016	NA	NA	SFXN4	Sign. RJF
CHR08FS021474416	21474416	chr8:21470578-21487179	3.47	0.035	NA	NA	KIF2C; SNORD38; RPS8	Sign. RJF
CHR11FS010792270	10792270	chr11:10782699-10793199	1.98	0.047	NA	NA	SLC7A10	Sign. RJF
CHR16FS000197847	197847	chr16:184547-208882	2.10	0.039	NA	NA	ZNF692; BG2; LOC769104	Sign. RJF
CHRZFS027972218	27972218	chrZ:27967699-27978199	-2.04	0.031	NA	NA	Novel gene in MHM	Sign. RJF
CHR01FS104511276	104511276	chr1:104503033-104513533	NA	NA	1.71	0.036		Sign. WL
CHR01FS112367091	112367091	chr1:112360548-112371048	NA	NA	-1.93	0.011	MX1	Sign. WL
CHR01FS171203823	171203823	chr1:171199298-171209798	NA	NA	-2.52	0.001	ENOX1	Sign. WL
CHR01FS017194209	17194209	chr1:17192667-17203167	NA	NA	-2.68	0.001	CERK; LOC770266	Sign. WL
CHR01FS189526406	189526406	chr1:189521776-189535485	NA	NA	-4.14	0.036	FAM76B; CEP57	Sign. WL
CHR01FS004027302	4027302	chr1:4022655-4033155	NA	NA	2.28	0.005	LOC419112; LOC776999	Sign. WL
CHR01FS055943153	55943153	chr1:55938503-55951519	NA	NA	2.55	0.001	CKAP4; TCP11L2	Sign. WL
CHR01FS087115614	87115614	chr1:87113677-87124177	NA	NA	3.02	0.005	ATP1B1	Sign. WL
CHR02FS023275619	23275619	chr2:23275059-23285559	NA	NA	-4.89	0.002	LOC420562	Sign. WL
CHR02FS044500430	44500430	chr2:44494000-44504500	NA	NA	2.55	0.001	CRTAP	Sign. WL
CHR02FS057276958	57276958	chr2:57271808-57282308	NA	NA	-2.52	0.024		Sign. WL
CHR02FS067096593	67096593	chr2:67086288-67096788	NA	NA	2.81	0.004	PECI; ECI2	Sign. WL
CHR02FS069054327	69054327	chr2:69053627-69064127	NA	NA	3.55	0.001	BCL2	Sign. WL
CHR21FS006498679	6498679	chr21:6498659-6509159	NA	NA	-3.16	0.002	WNT4	Sign. WL
CHR03FS069666761	69666761	chr3:69657369-69667869	NA	NA	-3.36	0.001	LOC421765	Sign. WL
CHR03FS074101495	74101495	chr3:74098092-74108592	NA	NA	-3.72	0.000	LOC421792	Sign. WL
CHR04FS065059350	65059350	chr4:65049419-65059919	NA	NA	-4.11	0.000	ZDHHC2	Sign. WL
CHR05FS028117494	28117494	chr5:28109050-28119550	NA	NA	-2.75	0.011	TTBK2	Sign. WL
CHR05FS062145487	62145487	chr5:62143821-62154321	NA	NA	3.43	0.014	LRFN5	Sign. WL
CHR06FS012548288	12548288	chr6:12539210-12552607	NA	NA	2.50	0.001	SGPL1; ANAPC16; ASCC1	Sign. WL
CHR12FS009183544	9183544	chr12:9174486-9184986	NA	NA	2.08	0.014	DNASE1L3	Sign. WL
CHR13FS001411354	1411354	chr13:1404838-1415338	NA	NA	2.20	0.011	PFDN1	Sign. WL
CHR15FS004985794	4985794	chr15:4977144-4991115	NA	NA	-2.25	0.020	TCTN2	Sign. WL
CHR18FS006303887	6303887	chr18:6296368-6306868	NA	NA	-2.68	0.041	COIL	Sign. WL
CHRZFS010859212	10859212	chrZ:10857968-10868468	NA	NA	-2.05	0.041	CZH5orf42	Sign. WL
CHRZFS027969552	27969552	chrZ:27967699-27978199	NA	NA	-2.37	0.002	Novel gene in MHM	Sign. WL
CHRZFS027969994	27969994	chrZ:27967699-27978199	NA	NA	-2.09	0.005	Novel gene in MHM	Sign. WL
CHRUNRFS020865610	20865610	chrUN_random:20863480-20873980	NA	NA	1.81	0.030	LOC425449; LOC426662	Sign. WL
CHRUNRFS020865792	20865792	chrUN_random:20863480-20873980	NA	NA	1.72	0.037	LOC425449; LOC426662	Sign. WL
CHRUNRFS022699787	22699787	chrUN_random:22699192-22709692	NA	NA	2.18	0.004	LOC429831; ZNF687	Sign. WL
CHRUNRFS022703457	22703457	chrUN_random:22699192-22709692	NA	NA	2.11	0.004	LOC429831; ZNF687	Sign. WL
CHRUNRFS022703739	22703739	chrUN_random:22699192-22709692	NA	NA	1.93	0.013	LOC429831; ZNF687	Sign. WL
CHRUNRFS047163489	47163489	chrUN_random:47158633-47169133	NA	NA	1.95	0.010	LOC430910; BTF3	Sign. WL
CHRUNRFS063230313	63230313	chrUN_random:63226657-63237157	NA	NA	1.82	0.040	AP2S1	Sign. WL

Genebuild: WASHUC2.
