# Supplementary material for: Large Sex Differences in Chicken Behavior and Brain Gene Expression Coincide with Few Differences in Promoter DNA-Methylation
Source: PLoS One. 2014 Apr 29;9(4):e96376. doi: 10.1371/journal.pone.0096376 (PMC4004567; doi:10.1371/journal.pone.0096376)
Supplement: Table S3 — Gene ontology analysis of significantly differentially expressed (DE) genes in females related to males. (PDF) [file pone.0096376.s004.pdf]

Table S3. Gene ontology analysis of significantly differentially expressed (DE) genes in females related to males, given as a 30 toplist within different categories.

				Occurence in		Significance	
**Category**	**GO ID**	**GO term**	**GO Level**	**Reference**	**Test list**	**Pval.**	**Pval. FDR-BH**
All DE genes across breeds (242 homologs found of 315 probesets included)							
	GO:0007166	cell surface receptor signaling pathway	3, 4, 5	2224	18	0.000	0.234
	GO:0023052	signaling	1	4227	47	0.000	0.215
	GO:0009892	negative regulation of metabolic process	2, 3, 4	1162	6	0.000	0.153
	GO:0006081	cellular aldehyde metabolic process	3	34	5	0.000	0.116
	GO:0001574	ganglioside biosynthetic process	7, 8, 9, 10	8	3	0.000	0.104
	GO:0007154	cell communication	2	4327	49	0.000	0.093
	GO:0006672	ceramide metabolic process	7, 8	62	6	0.000	0.153
	GO:0007186	G-protein coupled receptor signaling pathway	4, 5, 6	903	4	0.000	0.141
	GO:0031324	negative regulation of cellular metabolic process	3, 4, 5	1085	6	0.001	0.132
	GO:0007005	mitochondrion organization	5	177	10	0.001	0.124
	GO:0065007	biological regulation	1	7420	100	0.001	0.123
	GO:0000279	M phase	4, 5	477	18	0.001	0.114
	GO:0046519	sphingoid metabolic process	6, 7	66	6	0.001	0.114
	GO:0010605	negative regulation of macromolecule metabolic process	3, 4, 5	1057	6	0.001	0.118
	GO:0010165	response to X-ray	5	26	4	0.001	0.115
	GO:0045716	positive regulation of low-density lipoprotein particle receptor biosynthetic process	5, 6, 7, 8	3	2	0.001	0.114
	GO:0022403	cell cycle phase	3, 4	756	24	0.001	0.120
	GO:0071310	cellular response to organic substance	4	848	4	0.001	0.127
	GO:0000278	mitotic cell cycle	3	688	22	0.001	0.154
	GO:0007165	signal transduction	2, 3, 4	3830	45	0.001	0.146
	GO:0001573	ganglioside metabolic process	7, 8, 9	14	3	0.001	0.146
	GO:0006974	response to DNA damage stimulus	4	560	19	0.001	0.144
	GO:0009890	negative regulation of biosynthetic process	3, 4, 5	906	5	0.002	0.150
	GO:0051297	centrosome organization	5, 6, 8	53	5	0.002	0.146
	GO:0072369	regulation of lipid transport by positive regulation of transcription from RNA polymerase II promoter	6, 7, 8, 9, 10, 11, 12	4	2	0.002	0.144
	GO:0010988	regulation of low-density lipoprotein particle clearance	4, 5, 6	4	2	0.002	0.138
	GO:0006396	RNA processing	4, 5, 6, 7	569	19	0.002	0.138
	GO:0031327	negative regulation of cellular biosynthetic process	4, 5, 6	893	5	0.002	0.144
	GO:0033554	cellular response to stress	3	991	28	0.002	0.142
	GO:0006688	glycosphingolipid biosynthetic process	6, 7, 8	16	3	0.002	0.153
Higher DE genes across breeds (52 genes found of 67 probesets included)							
	GO:0035261	**external genitalia morphogenesis**	4, 5, 6, 7	6	2	0.000	0.163
	GO:0061010	gall bladder development	4, 5	6	2	0.000	0.082
	GO:0040018	positive regulation of multicellular organism growth	3, 4, 5	34	3	0.000	0.063
	GO:0034086	maintenance of sister chromatid cohesion	3, 4, 5, 7	8	2	0.000	0.076
	GO:0034088	maintenance of mitotic sister chromatid cohesion	4, 5, 6, 7, 8, 9, 10	8	2	0.000	0.061
	GO:0061038	**uterus morphogenesis**	3, 4, 5, 6	8	2	0.000	0.050
	GO:0060324	**face development**	3, 4	42	3	0.000	0.051
	GO:0071481	cellular response to X-ray	6	9	2	0.000	**0.049**
	GO:0035117	embryonic arm morphogenesis	7, 8	9	2	0.000	**0.043**
	GO:0035140	arm morphogenesis	6, 7	9	2	0.000	**0.039**
	GO:0007064	mitotic sister chromatid cohesion	4, 5, 6, 7, 8, 9	10	2	0.000	**0.044**
	GO:0031065	positive regulation of histone deacetylation	6, 7, 8, 9, 10, 11, 12	11	2	0.001	**0.049**
	GO:0090312	positive regulation of protein deacetylation	6, 7, 8, 9	13	2	0.001	0.064
	GO:0060322	**head development**	3	58	3	0.001	0.065
	GO:0048703	embryonic viscerocranium morphogenesis	4, 5, 7, 8, 9	14	2	0.001	0.064
	GO:0031063	regulation of histone deacetylation	6, 7, 8, 9, 11	15	2	0.001	0.069
	GO:0090311	regulation of protein deacetylation	6, 7, 8	17	2	0.001	0.084
	GO:0060065	**uterus development**	4, 5	18	2	0.002	0.089
	GO:0042634	regulation of hair cycle	3, 4	18	2	0.002	0.084
	GO:0035112	**genitalia morphogenesis**	3, 4, 5, 6	18	2	0.002	0.080
	GO:0040014	regulation of multicellular organism growth	3, 4	81	3	0.002	0.111
	GO:0032800	receptor biosynthetic process	4, 5	22	2	0.002	0.109
	GO:0048557	embryonic digestive tract morphogenesis	4, 5, 6, 7	24	2	0.003	0.123
	GO:0010165	response to X-ray	5	26	2	0.003	0.138
	GO:0031058	positive regulation of histone modification	5, 6, 7, 8, 9, 10, 11	28	2	0.004	0.124
	GO:0048562	embryonic organ morphogenesis	4, 5, 6	211	4	0.005	0.144
	GO:0035115	embryonic forelimb morphogenesis	6, 7	31	2	0.005	0.142
	GO:0007062	sister chromatid cohesion	3, 4, 6	31	2	0.005	0.138
	GO:0060325	face morphogenesis	3, 4, 5, 6	32	2	0.005	0.142
	GO:2001252	positive regulation of chromosome organization	4, 5, 6, 7	32	2	0.005	0.138
Lower DE genes across breeds (191 genes found of 248 probesets included)							
	GO:0065007	biological regulation	1	7420	73	0.000	0.097
	GO:0009892	negative regulation of metabolic process	2, 3, 4	1162	3	0.000	0.076
	GO:0031324	negative regulation of cellular metabolic process	3, 4, 5	1085	3	0.000	0.124
	GO:0010605	negative regulation of macromolecule metabolic process	3, 4, 5	1057	3	0.000	0.128
	GO:0009890	negative regulation of biosynthetic process	3, 4, 5	906	2	0.000	0.106
	GO:0007186	G-protein coupled receptor signaling pathway	4, 5, 6	903	2	0.000	0.094
	GO:0031327	negative regulation of cellular biosynthetic process	4, 5, 6	893	2	0.000	0.093
	GO:0007154	cell communication	2	4327	38	0.000	0.091
	GO:0023052	signaling	1	4227	37	0.000	0.091
	GO:0007005	mitochondrion organization	5	177	9	0.000	0.087
	GO:0006281	DNA repair	5, 6, 7	392	14	0.001	0.080
	GO:0009889	regulation of biosynthetic process	3, 4	2809	21	0.001	0.079
	GO:0010556	regulation of macromolecule biosynthetic process	4, 5	2615	19	0.001	0.080
	GO:0050794	regulation of cellular process	2, 3	6661	68	0.001	0.076
	GO:0051297	centrosome organization	5, 6, 8	53	5	0.001	0.072
	GO:0010468	regulation of gene expression	4, 5	2700	20	0.001	0.070
	GO:0050789	regulation of biological process	1, 2	7035	73	0.001	0.067
	GO:0031326	regulation of cellular biosynthetic process	4, 5	2785	21	0.001	0.066
	GO:0007166	cell surface receptor signaling pathway	3, 4, 5	2224	15	0.001	0.066
	GO:0010558	negative regulation of macromolecule biosynthetic process	4, 5, 6	821	2	0.001	0.075
	GO:0031023	microtubule organizing center organization	4, 5, 7	58	5	0.001	0.076
	GO:2000112	regulation of cellular macromolecule biosynthetic process	5, 6	2556	19	0.001	0.074
	GO:0006081	cellular aldehyde metabolic process	3	34	4	0.001	0.074
	GO:0006351	transcription, DNA-dependent	4, 5, 6, 7, 8	2458	18	0.001	0.072
	GO:2001141	regulation of RNA biosynthetic process	5, 6, 7, 8	2358	17	0.001	0.074
	GO:0032774	RNA biosynthetic process	4, 5, 6, 7	2529	19	0.001	0.077
	GO:0006355	regulation of transcription, DNA-dependent	5, 6, 7, 8, 9	2341	17	0.001	0.078
	GO:0006672	ceramide metabolic process	7, 8	62	5	0.001	0.079
	GO:0006364	rRNA processing	6, 7, 8, 9	94	6	0.001	0.083
	GO:2000113	negative regulation of cellular macromolecule biosynthetic process	5, 6, 7	781	2	0.001	0.083
All DE genes in RJF (213 homologs found of 276 probesets included)							
	GO:0009892	negative regulation of metabolic process	2, 3, 4	1162	4	0.000	0.199
	GO:0001574	ganglioside biosynthetic process	7, 8, 9, 10	8	3	0.000	0.171
	GO:0031324	negative regulation of cellular metabolic process	3, 4, 5	1085	4	0.000	0.169
	GO:0010605	negative regulation of macromolecule metabolic process	3, 4, 5	1057	4	0.000	0.176
	GO:0006396	RNA processing	4, 5, 6, 7	569	19	0.000	0.178

GO:0010165	response to X-ray	5	26	4	0.000	0.171
GO:0009890	negative regulation of biosynthetic process	3, 4, 5	906	3	0.001	0.160
GO:0007186	G-protein coupled receptor signaling pathway	4, 5, 6	903	3	0.001	0.145
GO:0007166	cell surface receptor signaling pathway	3, 4, 5	2224	17	0.001	0.138
GO:0031327	negative regulation of cellular biosynthetic process	4, 5, 6	893	3	0.001	0.131
GO:0045716	positive regulation of low-density lipoprotein particle receptor biosynthetic process	5, 6, 7, 8	3	2	0.001	0.123
GO:0007005	mitochondrion organization	5	177	9	0.001	0.159
GO:0051297	centrosome organization	5, 6, 8	53	5	0.001	0.149
GO:0001573	ganglioside metabolic process	7, 8, 9	14	3	0.001	0.146
GO:0010212	response to ionizing radiation	4	112	7	0.001	0.147
GO:0010988	regulation of low-density lipoprotein particle clearance	4, 5, 6	4	2	0.001	0.166
GO:0072369	regulation of lipid transport by positive regulation of transcription from RNA polymerase II promoter	6, 7, 8, 9, 10, 11, 12	4	2	0.001	0.156
GO:0006081	cellular aldehyde metabolic process	3	34	4	0.001	0.158
GO:0071479	cellular response to ionizing radiation	5	34	4	0.001	0.150
GO:0031023	microtubule organizing center organization	4, 5, 7	58	5	0.001	0.144
GO:0023052	signaling	1	4227	44	0.001	0.144
GO:0006688	glycosphingolipid biosynthetic process	6, 7, 8	16	3	0.001	0.139
GO:0010558	negative regulation of macromolecule biosynthetic process	4, 5, 6	821	3	0.001	0.134
GO:0006672	ceramide metabolic process	7, 8	62	5	0.002	0.160
GO:0007154	cell communication	2	4327	46	0.002	0.161
GO:0072367	regulation of lipid transport by regulation of transcription from RNA polymerase II promoter	5, 6, 7, 8, 9, 10, 11	5	2	0.002	0.168
GO:0006364	rRNA processing	6, 7, 8, 9	94	6	0.002	0.164
GO:2000113	negative regulation of cellular macromolecule biosynthetic process	5, 6, 7	781	3	0.002	0.175
GO:0006397	mRNA processing	5, 6, 7, 8	332	12	0.002	0.171
GO:0046519	sphingoid metabolic process	6, 7	66	5	0.002	0.167
Higher DE genes in RJF (38 genes found of 52 probesets included)						
GO:0035261	**external genitalia morphogenesis**	4, 5, 6, 7	6	2	0.000	0.069
GO:0061010	gall bladder development	4, 5	6	2	0.000	**0.035**
GO:0060324	face development	3, 4	42	3	0.000	**0.037**
GO:0034086	maintenance of sister chromatid cohesion	3, 4, 5, 7	8	2	0.000	**0.032**
GO:0061038	**uterus morphogenesis**	3, 4, 5, 6	8	2	0.000	**0.026**
GO:0034088	maintenance of mitotic sister chromatid cohesion	4, 5, 6, 7, 8, 9, 10	8	2	0.000	**0.021**
GO:0071481	cellular response to X-ray	6	9	2	0.000	**0.024**
GO:0035117	embryonic arm morphogenesis	7, 8	9	2	0.000	**0.021**
GO:0035140	arm morphogenesis	6, 7	9	2	0.000	**0.018**
GO:0007064	mitotic sister chromatid cohesion	4, 5, 6, 7, 8, 9	10	2	0.000	**0.021**
GO:0031065	positive regulation of histone deacetylation	6, 7, 8, 9, 10, 11, 12	11	2	0.000	**0.023**
GO:0060322	head development	3	58	3	0.000	**0.024**
GO:0090312	positive regulation of protein deacetylation	6, 7, 8, 9	13	2	0.000	**0.027**
GO:0048703	embryonic viscerocranium morphogenesis	4, 5, 7, 8, 9	14	2	0.000	**0.029**
GO:0031063	regulation of histone deacetylation	6, 7, 8, 9, 11	15	2	0.001	**0.032**
GO:0090311	regulation of protein deacetylation	6, 7, 8	17	2	0.001	**0.038**
GO:0060065	**uterus development**	4, 5	18	2	0.001	**0.040**
GO:0042634	regulation of hair cycle	3, 4	18	2	0.001	**0.038**
GO:0035112	**genitalia morphogenesis**	3, 4, 5, 6	18	2	0.001	**0.036**
GO:0032800	receptor biosynthetic process	4, 5	22	2	0.001	0.051
GO:0048562	embryonic organ morphogenesis	4, 5, 6	211	4	0.001	0.055
GO:0048557	embryonic digestive tract morphogenesis	4, 5, 6, 7	24	2	0.001	0.056
GO:0010165	response to X-ray	5	26	2	0.002	0.062
GO:0031058	positive regulation of histone modification	5, 6, 7, 8, 9, 10, 11	28	2	0.002	0.069
GO:0007548	**sex differentiation**	3, 4	244	4	0.002	0.078
GO:0042471	ear morphogenesis	5, 6, 7	114	3	0.002	0.075
GO:0007062	sister chromatid cohesion	3, 4, 6	31	2	0.002	0.063
GO:0035115	embryonic forelimb morphogenesis	6, 7	31	2	0.002	0.061
GO:0060325	face morphogenesis	3, 4, 5, 6	32	2	0.003	0.063
GO:2001252	positive regulation of chromosome organization	4, 5, 6, 7	32	2	0.003	0.062
Lower DE genes in RJF (176 genes found of 224 probesets included)						
GO:0009892	negative regulation of metabolic process	2, 3, 4	1162	2	0.000	0.078
GO:0031324	negative regulation of cellular metabolic process	3, 4, 5	1085	2	0.000	0.094
GO:0010605	negative regulation of macromolecule metabolic process	3, 4, 5	1057	2	0.000	0.065
GO:0065007	biological regulation	1	7420	69	0.000	0.066
GO:0051297	centrosome organization	5, 6, 8	53	5	0.000	0.105
GO:0010556	regulation of macromolecule biosynthetic process	4, 5	2615	17	0.001	0.122
GO:0031023	microtubule organizing center organization	4, 5, 7	58	5	0.001	0.105
GO:0009889	regulation of biosynthetic process	3, 4	2809	19	0.001	0.097
GO:0007186	G-protein coupled receptor signaling pathway	4, 5, 6	903	2	0.001	0.102
GO:0031326	regulation of cellular biosynthetic process	4, 5	2785	19	0.001	0.100
GO:0006364	rRNA processing	6, 7, 8, 9	94	6	0.001	0.110
GO:2000112	regulation of cellular macromolecule biosynthetic process	5, 6	2556	17	0.001	0.109
GO:0042254	ribosome biogenesis	5	131	7	0.001	0.104
GO:0007154	cell communication	2	4327	36	0.001	0.100
GO:0023052	signaling	1	4227	35	0.001	0.101
GO:0006396	RNA processing	4, 5, 6, 7	569	16	0.001	0.097
GO:0007005	mitochondrion organization	5	177	8	0.001	0.103
GO:0016072	rRNA metabolic process	6, 7, 8	101	6	0.001	0.105
GO:0050794	regulation of cellular process	2, 3	6661	64	0.001	0.114
GO:0051172	negative regulation of nitrogen compound metabolic process	3, 4, 5	827	2	0.002	0.119
GO:0045934	negative regulation of nucleobase-containing compound metabolic process	4, 5, 6	819	2	0.002	0.124
GO:0022900	electron transport chain	3, 4	147	7	0.002	0.122
GO:0050789	regulation of biological process	1, 2	7035	69	0.002	0.120
GO:0006281	DNA repair	5, 6, 7	392	12	0.002	0.139
GO:0007166	cell surface receptor signaling pathway	3, 4, 5	2224	15	0.002	0.143
GO:0010468	regulation of gene expression	4, 5	2700	20	0.002	0.147
GO:0032502	developmental process	1	3808	32	0.002	0.143
GO:0018130	heterocycle biosynthetic process	4	355	11	0.003	0.167
GO:0070841	inclusion body assembly	5	7	2	0.003	0.163
GO:0022403	cell cycle phase	3, 4	756	18	0.003	0.160
All DE genes in WL (165 homologs found of 204 probesets included)						
GO:0007166	cell surface receptor signaling pathway	3, 4, 5	2224	7	0.000	**0.008**
GO:0065007	biological regulation	1	7420	58	0.000	**0.012**
GO:0050794	regulation of cellular process	2, 3	6661	53	0.000	0.068
GO:0050789	regulation of biological process	1, 2	7035	58	0.000	0.085
GO:0007154	cell communication	2	4327	30	0.000	0.091
GO:0045716	positive regulation of low-density lipoprotein particle receptor biosynthetic process	5, 6, 7, 8	3	2	0.000	0.093
GO:0023052	signaling	1	4227	30	0.000	0.105
GO:0071479	cellular response to ionizing radiation	5	34	4	0.001	0.099
GO:0006081	cellular aldehyde metabolic process	3	34	4	0.001	0.089
GO:0000086	G2/M transition of mitotic cell cycle	3, 4, 5, 6, 7	135	7	0.001	0.109
GO:0072369	regulation of lipid transport by positive regulation of transcription from RNA polymerase II promoter	6, 7, 8, 9, 10, 11, 12	4	2	0.001	0.107
GO:0010988	regulation of low-density lipoprotein particle clearance	4, 5, 6	4	2	0.001	0.099
GO:0006974	response to DNA damage stimulus	4	560	15	0.001	0.131
GO:0072367	regulation of lipid transport by regulation of transcription from RNA polymerase II promoter	5, 6, 7, 8, 9, 10, 11	5	2	0.001	0.141
GO:0022403	cell cycle phase	3, 4	756	18	0.001	0.137
GO:0010212	response to ionizing radiation	4	112	6	0.001	0.142
GO:0010984	regulation of lipoprotein particle clearance	3, 4, 5	6	2	0.002	0.175
GO:0061010	gall bladder development	4, 5	6	2	0.002	0.166

GO:0035261	**external genitalia morphogenesis**	4, 5, 6, 7	6	2	0.002	0.157
GO:0033554	cellular response to stress	3	991	21	0.002	0.167
GO:0048583	regulation of response to stimulus	2, 3	1881	10	0.002	0.163
GO:0046487	glyoxylate metabolic process	4, 7	7	2	0.002	0.189
GO:0045714	regulation of low-density lipoprotein particle receptor biosynthetic process	5, 6, 7	7	2	0.002	0.181
GO:0070841	inclusion body assembly	5	7	2	0.002	0.174
GO:0007165	signal transduction	2, 3, 4	3830	29	0.003	0.171
GO:0009889	regulation of biosynthetic process	3, 4	2809	19	0.003	0.176
GO:0000278	mitotic cell cycle	3	688	16	0.003	0.172
GO:0010468	regulation of gene expression	4, 5	2700	18	0.003	0.168
GO:0010165	response to X-ray	5	26	3	0.003	0.163
GO:0042254	ribosome biogenesis	5	131	6	0.003	0.165
Higher DE genes in WL (32 genes found of 41 probesets included)						
GO:0040018	positive regulation of multicellular organism growth	3, 4, 5	34	3	0.000	**0.026**
GO:0061010	gall bladder development	4, 5	6	2	0.000	**0.019**
GO:0035261	**external genitalia morphogenesis**	4, 5, 6, 7	6	2	0.000	**0.013**
GO:0034086	maintenance of sister chromatid cohesion	3, 4, 5, 7	8	2	0.000	**0.018**
GO:0061038	**uterus morphogenesis**	3, 4, 5, 6	8	2	0.000	**0.014**
GO:0034088	maintenance of mitotic sister chromatid cohesion	4, 5, 6, 7, 8, 9, 10	8	2	0.000	**0.012**
GO:0071481	cellular response to X-ray	6	9	2	0.000	**0.013**
GO:0035117	embryonic arm morphogenesis	7, 8	9	2	0.000	**0.011**
GO:0035140	arm morphogenesis	6, 7	9	2	0.000	**0.010**
GO:0007064	mitotic sister chromatid cohesion	4, 5, 6, 7, 8, 9	10	2	0.000	**0.011**
GO:0031065	positive regulation of histone deacetylation	6, 7, 8, 9, 10, 11, 12	11	2	0.000	**0.013**
GO:0090312	positive regulation of protein deacetylation	6, 7, 8, 9	13	2	0.000	**0.016**
GO:0048703	embryonic viscerocranium morphogenesis	4, 5, 7, 8, 9	14	2	0.000	**0.017**
GO:0031063	regulation of histone deacetylation	6, 7, 8, 9, 11	15	2	0.000	**0.019**
GO:0090311	regulation of protein deacetylation	6, 7, 8	17	2	0.000	**0.023**
GO:0040014	regulation of multicellular organism growth	3, 4	81	3	0.000	**0.021**
GO:0060065	**uterus development**	4, 5	18	2	0.001	**0.022**
GO:0035112	**genitalia morphogenesis**	3, 4, 5, 6	18	2	0.001	**0.021**
GO:0042634	regulation of hair cycle	3, 4	18	2	0.001	**0.020**
GO:0048557	embryonic digestive tract morphogenesis	4, 5, 6, 7	24	2	0.001	**0.034**
GO:0010165	response to X-ray	5	26	2	0.001	**0.038**
GO:0048638	regulation of developmental growth	3, 4	110	3	0.001	**0.037**
GO:0031058	positive regulation of histone modification	5, 6, 7, 8, 9, 10, 11	28	2	0.001	**0.040**
GO:0007062	sister chromatid cohesion	3, 4, 6	31	2	0.002	**0.047**
GO:0035115	embryonic forelimb morphogenesis	6, 7	31	2	0.002	**0.045**
GO:2001252	positive regulation of chromosome organization	4, 5, 6, 7	32	2	0.002	**0.046**
GO:0060325	**face morphogenesis**	3, 4, 5, 6	32	2	0.002	**0.044**
GO:0000279	M phase	4, 5	477	5	0.002	**0.044**
GO:0035264	multicellular organism growth	2	131	3	0.002	**0.046**
GO:0071479	cellular response to ionizing radiation	5	34	2	0.002	**0.045**
GO:0016575	histone deacetylation	7, 8, 10	35	2	0.002	**0.045**
GO:0048701	**embryonic cranial skeleton morphogenesis**	6, 7, 8	37	2	0.002	**0.048**
GO:0060323	**head morphogenesis**	3, 4, 5	38	2	0.002	**0.049**
GO:0045927	positive regulation of growth	2, 3, 4	143	3	0.002	**0.049**
Lower DE genes in RJF (134 genes found of 163 probesets included)						
GO:0065007	biological regulation	1	7420	43	0.000	**0.002**
GO:0050794	regulation of cellular process	2, 3	6661	39	0.000	**0.009**
GO:0050789	regulation of biological process	1, 2	7035	43	0.000	**0.010**
GO:0007166	cell surface receptor signaling pathway	3, 4, 5	2224	6	0.000	**0.015**
GO:0006081	cellular aldehyde metabolic process	3	34	4	0.000	0.083
GO:0010468	regulation of gene expression	4, 5	2700	11	0.000	0.073
GO:0009889	regulation of biosynthetic process	3, 4	2809	12	0.000	0.076
GO:0007154	cell communication	2	4327	24	0.000	0.078
GO:0031326	regulation of cellular biosynthetic process	4, 5	2785	12	0.000	0.069
GO:0048583	regulation of response to stimulus	2, 3	1881	6	0.001	0.081
GO:0031323	regulation of cellular metabolic process	3, 4	3653	19	0.001	0.076
GO:0023052	signaling	1	4227	24	0.001	0.089
GO:0006281	DNA repair	5, 6, 7	392	11	0.001	0.102
GO:0019222	regulation of metabolic process	2, 3	4057	23	0.001	0.099
GO:0080090	regulation of primary metabolic process	3, 4	3560	19	0.001	0.094
GO:0051171	regulation of nitrogen compound metabolic process	3, 4	2908	14	0.001	0.094
GO:2001141	regulation of RNA biosynthetic process	5, 6, 7, 8	2358	10	0.001	0.096
GO:0010556	regulation of macromolecule biosynthetic process	4, 5	2615	12	0.001	0.099
GO:0006355	regulation of transcription, DNA-dependent	5, 6, 7, 8, 9	2341	10	0.001	0.095
GO:0042254	ribosome biogenesis	5	131	6	0.001	0.098
GO:0032501	multicellular organismal process	1	4702	29	0.001	0.095
GO:0006351	transcription, DNA-dependent	4, 5, 6, 7, 8	2458	11	0.001	0.092
GO:0000086	G2/M transition of mitotic cell cycle	3, 4, 5, 6, 7	135	6	0.001	0.099
GO:0019219	regulation of nucleobase-containing compound metabolic process	4, 5	2832	14	0.001	0.097
GO:2000112	regulation of cellular macromolecule biosynthetic process	5, 6	2556	12	0.002	0.097
GO:0006364	rRNA processing	6, 7, 8, 9	94	5	0.002	0.097
GO:0032502	developmental process	1	3808	22	0.002	0.095
GO:0006396	RNA processing	4, 5, 6, 7	569	13	0.002	0.096
GO:0051252	regulation of RNA metabolic process	4, 5, 6, 7	2404	11	0.002	0.093
GO:0070841	inclusion body assembly	5	7	2	0.002	0.090

Analysis was performed with human homologs using the Manteia web tool.
