# Supplementary material for: Large Sex Differences in Chicken Behavior and Brain Gene Expression Coincide with Few Differences in Promoter DNA-Methylation
Source: PLoS One. 2014 Apr 29;9(4):e96376. doi: 10.1371/journal.pone.0096376 (PMC4004567; doi:10.1371/journal.pone.0096376)
Supplement: Table S4 — Gene ontology analysis of significantly differentially methylated (DM) promoters in females related to males. (PDF) [file pone.0096376.s005.pdf]

Table S4. Gene ontology analysis of significantly differentially methylated (DM) promoters in females related to males, given as a 30 toplist within different categories.

Category	GO ID	GO term	GO Level	Occurence in Reference	Occurence in Test list	Significance Pval.	Significance Pval. FDR-BH
All DM promoters across breeds (30 homologs found of 55 loci included)							
	GO:0018345	protein palmitoylation	7	10	2	0.000	0.204
	GO:0009615	response to virus	3, 4	200	4	0.001	0.381
	GO:0007281	germ cell development	3, 4, 5	133	3	0.002	0.128
	GO:0009994	**oocyte differentiation**	3, 4, 6, 7	36	2	0.002	0.124
	GO:0048599	**oocyte development**	4, 5, 6, 7, 8	36	2	0.002	0.118
	GO:0009607	response to biotic stimulus	2	485	5	0.003	0.119
	GO:0008637	apoptotic mitochondrial changes	5, 6	40	2	0.003	0.134
	GO:0016044	cellular membrane organization	2, 4	320	4	0.004	0.156
	GO:0061024	membrane organization	3	322	4	0.004	0.153
	GO:0006497	protein lipidation	6	46	2	0.004	0.156
	GO:0001658	**branching involved in ureteric bud morphogenesis**	4, 5, 6, 7, 8	49	2	0.005	0.118
	GO:0002376	immune system process	1	1383	8	0.005	0.116
	GO:0042158	lipoprotein biosynthetic process	5	50	2	0.005	0.117
	GO:0060675	**ureteric bud morphogenesis**	5, 6, 7	56	2	0.006	0.142
	GO:0048477	**oogenesis**	5, 6	57	2	0.006	0.144
	GO:0006921	cellular component disassembly involved in apoptotic process	5	68	2	0.008	0.142
	GO:0007276	**gamete generation**	3, 4	415	4	0.009	0.146
	GO:0033077	T cell differentiation in thymus	6, 7, 8, 9, 10	71	2	0.009	0.149
	GO:0032501	multicellular organismal process	1	4702	16	0.009	0.152
	GO:0072006	nephron development	3, 5, 6, 7	73	2	0.010	0.153
	GO:0022037	metencephalon development	3, 4, 6, 7, 8	74	2	0.010	0.155
	GO:0071845	cellular component disassembly at cellular level	4	227	3	0.010	0.159
	GO:0042157	lipoprotein metabolic process	4	77	2	0.011	0.140
	GO:0022411	cellular component disassembly	3	230	3	0.011	0.139
	GO:0001656	metanephros development	5, 6, 7	80	2	0.011	0.147
	GO:0051707	response to other organism	2, 3	460	4	0.012	0.156
	GO:0019953	**sexual reproduction**	2	469	4	0.013	0.143
	GO:0016050	vesicle organization	5	86	2	0.013	0.142
	GO:0007292	**female gamete generation**	4, 5	87	2	0.013	0.143
	GO:0008543	fibroblast growth factor receptor signaling pathway	5, 6, 7, 8	89	2	0.014	0.148
Hypo DM promoters across breeds (8 homologs found of 24 loci included)							
	GO:0018345	protein palmitoylation	7	10	2	0.000	**0.018**
	GO:0006497	protein lipidation	6	46	2	0.001	0.205
	GO:0042158	lipoprotein biosynthetic process	5	50	2	0.001	0.162
	GO:0042157	lipoprotein metabolic process	4	77	2	0.002	**0.047**
	GO:0008543	fibroblast growth factor receptor signaling pathway	5, 6, 7, 8	89	2	0.003	0.060
	GO:0071774	response to fibroblast growth factor stimulus	3, 5	105	2	0.003	0.058
	GO:0044344	cellular response to fibroblast growth factor stimulus	4, 6	105	2	0.003	0.056
	GO:0043543	protein acylation	6	123	2	0.005	0.063
	GO:0007281	germ cell development	3, 4, 5	133	2	0.005	0.061
	GO:0008219	cell death	2	1548	5	0.006	0.065
	GO:0016265	death	1	1551	5	0.006	0.064
	GO:0009607	response to biotic stimulus	2	485	3	0.007	0.067
	GO:0009615	response to virus	3, 4	200	2	0.012	0.082
	GO:0071363	cellular response to growth factor stimulus	5	212	2	0.013	0.087
	GO:0070848	response to growth factor stimulus	4	226	2	0.015	0.090
	GO:0022607	cellular component assembly	3	1275	4	0.016	0.094
	GO:0044085	cellular component biogenesis	2	1394	4	0.022	0.110
	GO:0016044	cellular membrane organization	2, 4	320	2	0.028	0.124
	GO:0061024	membrane organization	3	322	2	0.028	0.121
	GO:0071310	cellular response to organic substance	4	848	3	0.028	0.122
	GO:0051704	multi-organism process	1	870	3	0.030	0.128
	GO:0071844	cellular component assembly at cellular level	4	940	3	0.036	0.140
	GO:0055114	oxidation-reduction process	2	946	3	0.037	0.140
	GO:0003006	**developmental process involved in reproduction**	2, 3	402	2	0.041	0.147
	GO:0007276	**gamete generation**	3, 4	415	2	0.044	0.152
	GO:0045087	innate immune response	3, 4	441	2	0.048	0.157
	GO:0051707	response to other organism	2, 3	460	2	0.052	0.165
	GO:0019953	**sexual reproduction**	2	469	2	0.053	0.168
	GO:0006184	GTP catabolic process	9, 10, 11, 12	470	2	0.054	0.168
	GO:0046039	GTP metabolic process	8, 9, 10, 11	482	2	0.056	0.169
Hyper DM promoters across breeds (18 homologs found of 31 loci included)							
	GO:0032501	multicellular organismal process	1	4702	13	0.000	0.277
	GO:0022037	metencephalon development	3, 4, 6, 7, 8	74	2	0.004	0.214
	GO:0016050	vesicle organization	5	86	2	0.005	0.248
	GO:0001701	in utero embryonic development	6	308	3	0.005	0.185
	GO:0030902	hindbrain development	3, 5, 6, 7	112	2	0.008	0.186
	GO:0051607	defense response to virus	3, 4, 5	120	2	0.009	0.159
	GO:0007275	multicellular organismal development	2	3449	9	0.009	0.156
	GO:0042113	B cell activation	4, 5	146	2	0.013	0.177
	GO:0032502	developmental process	1	3808	9	0.016	0.213
	GO:0002376	immune system process	1	1383	5	0.017	0.203
	GO:0043009	chordate embryonic development	5	499	3	0.019	0.219
	GO:0009792	embryo development ending in birth or egg hatching	4	511	3	0.020	0.222
	GO:0055085	transmembrane transport	2, 3, 4	944	4	0.020	0.223
	GO:0006811	ion transport	3, 4	945	4	0.020	0.220
	GO:0034645	cellular macromolecule biosynthetic process	4	3279	8	0.021	0.219
	GO:0009615	response to virus	3, 4	200	2	0.023	0.229
	GO:0009059	macromolecule biosynthetic process	3	3354	8	0.023	0.225
	GO:0001666	response to hypoxia	3, 4	206	2	0.024	0.227
	GO:0050900	leukocyte migration	2, 4, 5	209	2	0.025	0.220
	GO:0034641	cellular nitrogen compound metabolic process	3	4837	10	0.026	0.213
	GO:0044249	cellular biosynthetic process	3	4123	9	0.026	0.214
	GO:0010467	gene expression	3	3426	8	0.026	0.212
	GO:0070482	response to oxygen levels	3	222	2	0.027	0.215

	GO:0040011	locomotion	1	1048	4	0.028	0.215
	GO:0071845	cellular component disassembly at cellular level	4	227	2	0.028	0.217
	GO:0006807	nitrogen compound metabolic process	2	4924	10	0.029	0.217
	GO:0022411	cellular component disassembly	3	230	2	0.029	0.208
	GO:0009058	biosynthetic process	2	4217	9	0.030	0.210
	GO:0090304	nucleic acid metabolic process	4, 5	3542	8	0.031	0.210
	GO:0006139	nucleobase-containing compound metabolic process	3, 4	4432	9	0.039	0.218
All DM promoters in RJF(12 homologs found of 22 loci included)							
	GO:0001701	in utero embryonic development	6	308	3	0.002	0.118
	GO:0032501	multicellular organismal process	1	4702	9	0.004	0.135
	GO:0043009	chordate embryonic development	5	499	3	0.007	0.121
	GO:0009792	embryo development ending in birth or egg hatching	4	511	3	0.008	0.118
	GO:0009790	embryo development	3	854	3	0.029	0.249
	GO:0055085	transmembrane transport	2, 3, 4	944	3	0.037	0.277
	GO:0006811	ion transport	3, 4	945	3	0.037	0.266
	GO:0032774	RNA biosynthetic process	4, 5, 6, 7	2529	5	0.039	0.271
	GO:0007275	multicellular organismal development	2	3449	6	0.039	0.263
	GO:0006366	transcription from RNA polymerase II promoter	5, 6, 7, 8, 9	1100	3	0.052	0.314
	GO:0032502	developmental process	1	3808	6	0.058	0.326
	GO:0016070	RNA metabolic process	4, 5, 6	3071	5	0.073	0.339
	GO:0065008	regulation of biological quality	2	2200	4	0.079	0.339
	GO:0034645	cellular macromolecule biosynthetic process	4	3279	5	0.088	0.334
	GO:0006355	regulation of transcription, DNA-dependent	5, 6, 7, 8, 9	2341	4	0.091	0.331
	GO:2001141	regulation of RNA biosynthetic process	5, 6, 7, 8	2358	4	0.093	0.330
	GO:0009059	macromolecule biosynthetic process	3	3354	5	0.094	0.328
	GO:0051252	regulation of RNA metabolic process	4, 5, 6, 7	2404	4	0.097	0.326
	GO:0010467	gene expression	3	3426	5	0.100	0.329
	GO:0048878	chemical homeostasis	4	700	2	0.100	0.318
	GO:0006351	transcription, DNA-dependent	4, 5, 6, 7, 8	2458	4	0.102	0.299
	GO:0006812	cation transport	4, 5	726	2	0.106	0.299
	GO:0090304	nucleic acid metabolic process	4, 5	3542	5	0.109	0.299
	GO:2000112	regulation of cellular macromolecule biosynthetic process	5, 6	2556	4	0.112	0.298
	GO:0010556	regulation of macromolecule biosynthetic process	4, 5	2615	4	0.118	0.295
	GO:0050896	response to stimulus	1	5834	3	0.121	0.299
	GO:0010468	regulation of gene expression	4, 5	2700	4	0.126	0.299
	GO:0034641	cellular nitrogen compound metabolic process	3	4837	6	0.127	0.298
	GO:0051716	cellular response to stimulus	2	4600	2	0.129	0.296
	GO:0006807	nitrogen compound metabolic process	2	4924	6	0.133	0.297
Hypo DM promoters in RJF (3 homologs found of 9 loci included)*							
	GO:0048878	chemical homeostasis	4	700	2	0.007	0.087
	GO:0042592	homeostatic process	3	1010	2	0.015	0.110
	GO:0065008	regulation of biological quality	2	2200	2	0.063	0.190
	GO:0065007	biological regulation	1	7420	2	0.399	0.521
	GO:0009987	cellular process	1	10797	2	0.403	0.514
	GO:0008152	metabolic process	1	8358	2	0.433	0.472
Hyper DM promoter in RJF (9 homologs found of 13 loci included)							
	GO:0001701	in utero embryonic development	6	308	3	0.001	0.058
	GO:0032501	multicellular organismal process	1	4702	8	0.001	**0.050**
	GO:0043009	chordate embryonic development	5	499	3	0.003	0.076
	GO:0009792	embryo development ending in birth or egg hatching	4	511	3	0.003	0.061
	GO:0007275	multicellular organismal development	2	3449	6	0.008	0.094
	GO:0032774	RNA biosynthetic process	4, 5, 6, 7	2529	5	0.011	0.110
	GO:0009790	embryo development	3	854	3	0.013	0.115
	GO:0032502	developmental process	1	3808	6	0.014	0.105
	GO:0016070	RNA metabolic process	4, 5, 6	3071	5	0.025	0.163
	GO:0006366	transcription from RNA polymerase II promoter	5, 6, 7, 8, 9	1100	3	0.025	0.162
	GO:0034645	cellular macromolecule biosynthetic process	4	3279	5	0.032	0.184
	GO:0009059	macromolecule biosynthetic process	3	3354	5	0.034	0.193
	GO:0010467	gene expression	3	3426	5	0.037	0.201
	GO:0006355	regulation of transcription, DNA-dependent	5, 6, 7, 8, 9	2341	4	0.041	0.211
	GO:2001141	regulation of RNA biosynthetic process	5, 6, 7, 8	2358	4	0.041	0.208
	GO:0034641	cellular nitrogen compound metabolic process	3	4837	6	0.042	0.203
	GO:0090304	nucleic acid metabolic process	4, 5	3542	5	0.042	0.198
	GO:0051252	regulation of RNA metabolic process	4, 5, 6, 7	2404	4	0.044	0.194
	GO:0006807	nitrogen compound metabolic process	2	4924	6	0.045	0.193
	GO:0006351	transcription, DNA-dependent	4, 5, 6, 7, 8	2458	4	0.047	0.195
	GO:2000112	regulation of cellular macromolecule biosynthetic process	5, 6	2556	4	0.052	0.207
	GO:0010556	regulation of macromolecule biosynthetic process	4, 5	2615	4	0.056	0.215
	GO:0010468	regulation of gene expression	4, 5	2700	4	0.061	0.213
	GO:0031326	regulation of cellular biosynthetic process	4, 5	2785	4	0.067	0.216
	GO:0009889	regulation of biosynthetic process	3, 4	2809	4	0.068	0.217
	GO:0019219	regulation of nucleobase-containing compound metabolic process	4, 5	2832	4	0.070	0.212
	GO:0044249	cellular biosynthetic process	3	4123	5	0.071	0.212
	GO:0051171	regulation of nitrogen compound metabolic process	3, 4	2908	4	0.075	0.219
	GO:0009058	biosynthetic process	2	4217	5	0.077	0.220
	GO:0006139	nucleobase-containing compound metabolic process	3, 4	4432	5	0.090	0.212
All DM promoters in WL (12 homologs found of 22 loci included)							
	GO:0018345	protein palmitoylation	7	10	2	0.000	0.121
	GO:0009615	response to virus	3, 4	200	4	0.000	0.146
	GO:0007281	germ cell development	3, 4, 5	133	3	0.001	0.435
	GO:0048599	**oocyte development**	4, 5, 6, 7, 8	36	2	0.002	0.406
	GO:0009994	**oocyte differentiation**	3, 4, 6, 7	36	2	0.002	0.325
	GO:0016044	cellular membrane organization	2, 4	320	4	0.002	0.273
	GO:0061024	membrane organization	3	322	4	0.002	0.239
	GO:0008637	apoptotic mitochondrial changes	5, 6	40	2	0.002	0.080
	GO:0006497	protein lipidation	6	46	2	0.003	0.101
	GO:0001658	branching involved in ureteric bud morphogenesis	4, 5, 6, 7, 8	49	2	0.003	0.110
	GO:0042158	lipoprotein biosynthetic process	5	50	2	0.003	0.110
	GO:0060675	**ureteric bud morphogenesis**	5, 6, 7	56	2	0.004	0.094
	GO:0048477	**oogenesis**	5, 6	57	2	0.004	0.095

GO:0007276	**gamete generation**	3, 4	415	4	0.004	0.095
GO:0002376	immune system process	1	1383	7	0.004	0.104
GO:0006921	cellular component disassembly involved in apoptotic process	5	68	2	0.005	0.106
GO:0051707	response to other organism	2, 3	460	4	0.006	0.107
GO:0033077	T cell differentiation in thymus	6, 7, 8, 9, 10	71	2	0.006	0.110
GO:0019953	**sexual reproduction**	2	469	4	0.006	0.110
GO:0072006	nephron development	3, 5, 6, 7	73	2	0.006	0.112
GO:0022037	metencephalon development	3, 4, 6, 7, 8	74	2	0.006	0.113
GO:0009607	response to biotic stimulus	2	485	4	0.007	0.101
GO:0042157	lipoprotein metabolic process	4	77	2	0.007	0.103
GO:0032502	developmental process	1	3808	12	0.007	0.106
GO:0001656	metanephros development	5, 6, 7	80	2	0.007	0.107
GO:0016050	vesicle organization	5	86	2	0.008	0.106
GO:0007292	**female gamete generation**	4, 5	87	2	0.009	0.107
GO:0008543	fibroblast growth factor receptor signaling pathway	5, 6, 7, 8	89	2	0.009	0.111
GO:0001657	**ureteric bud development**	4	90	2	0.009	0.112
GO:0008584	**male gonad development**	5, 6, 7	91	2	0.009	0.113
Hypo DM promoters in WL (9 homologs found of 16 loci included)						
GO:0018345	protein palmitoylation	7	10	2	0.000	**0.010**
GO:0006497	protein lipidation	6	46	2	0.000	0.108
GO:0042158	lipoprotein biosynthetic process	5	50	2	0.000	0.085
GO:0042157	lipoprotein metabolic process	4	77	2	0.001	**0.035**
GO:0008543	fibroblast growth factor receptor signaling pathway	5, 6, 7, 8	89	2	0.001	**0.032**
GO:0044344	cellular response to fibroblast growth factor stimulus	4, 6	105	2	0.002	**0.042**
GO:0071774	response to fibroblast growth factor stimulus	3, 5	105	2	0.002	**0.041**
GO:0043543	protein acylation	6	123	2	0.003	**0.040**
GO:0007281	germ cell development	3, 4, 5	133	2	0.003	**0.045**
GO:0009615	response to virus	3, 4	200	2	0.007	0.057
GO:0071363	cellular response to growth factor stimulus	5	212	2	0.008	0.057
GO:0070848	response to growth factor stimulus	4	226	2	0.008	0.058
GO:0008219	cell death	2	1548	4	0.011	0.067
GO:0016265	death	1	1551	4	0.011	0.067
GO:0071310	cellular response to organic substance	4	848	3	0.013	0.072
GO:0051704	multi-organism process	1	870	3	0.014	0.073
GO:0016044	cellular membrane organization	2, 4	320	2	0.016	0.081
GO:0061024	membrane organization	3	322	2	0.016	0.081
GO:0003006	developmental process involved in reproduction	2, 3	402	2	0.025	0.100
GO:0007276	gamete generation	3, 4	415	2	0.026	0.101
GO:0070887	cellular response to chemical stimulus	3	1150	3	0.028	0.108
GO:0045087	innate immune response	3, 4	441	2	0.029	0.108
GO:0051707	response to other organism	2, 3	460	2	0.031	0.110
GO:0019953	sexual reproduction	2	469	2	0.032	0.111
GO:0006184	GTP catabolic process	9, 10, 11, 12	470	2	0.032	0.111
GO:0046039	GTP metabolic process	8, 9, 10, 11	482	2	0.034	0.115
GO:0009607	response to biotic stimulus	2	485	2	0.034	0.113
GO:0071495	cellular response to endogenous stimulus	3	487	2	0.035	0.114
GO:0007166	cell surface receptor signaling pathway	3, 4, 5	2224	4	0.035	0.112
GO:0048610	cellular process involved in reproduction	2, 3	496	2	0.036	0.114
Hyper DM promoters in WL (15 homologs found of 23 loci included)						
GO:0022037	metencephalon development	3, 4, 6, 7, 8	74	2	0.002	0.178
GO:0001701	in utero embryonic development	6	308	3	0.003	0.191
GO:0016050	vesicle organization	5	86	2	0.003	0.183
GO:0032501	multicellular organismal process	1	4702	10	0.004	0.202
GO:0030902	hindbrain development	3, 5, 6, 7	112	2	0.005	0.165
GO:0051607	defense response to virus	3, 4, 5	120	2	0.006	0.156
GO:0007275	multicellular organismal development	2	3449	8	0.008	0.153
GO:0042113	B cell activation	4, 5	146	2	0.009	0.164
GO:0043009	chordate embryonic development	5	499	3	0.011	0.164
GO:0009792	embryo development ending in birth or egg hatching	4	511	3	0.012	0.168
GO:0032502	developmental process	1	3808	8	0.014	0.174
GO:0009615	response to virus	3, 4	200	2	0.016	0.192
GO:0001666	response to hypoxia	3, 4	206	2	0.017	0.197
GO:0070482	response to oxygen levels	3	222	2	0.019	0.206
GO:0034645	cellular macromolecule biosynthetic process	4	3279	7	0.021	0.201
GO:0044249	cellular biosynthetic process	3	4123	8	0.022	0.199
GO:2000112	regulation of cellular macromolecule biosynthetic process	5, 6	2556	6	0.023	0.203
GO:0009059	macromolecule biosynthetic process	3	3354	7	0.024	0.196
GO:0009058	biosynthetic process	2	4217	8	0.025	0.194
GO:0010556	regulation of macromolecule biosynthetic process	4, 5	2615	6	0.025	0.195
GO:0010467	gene expression	3	3426	7	0.026	0.199
GO:0010468	regulation of gene expression	4, 5	2700	6	0.029	0.189
GO:0090304	nucleic acid metabolic process	4, 5	3542	7	0.031	0.192
GO:0006139	nucleobase-containing compound metabolic process	3, 4	4432	8	0.032	0.196
GO:0031326	regulation of cellular biosynthetic process	4, 5	2785	6	0.033	0.197
GO:0009889	regulation of biosynthetic process	3, 4	2809	6	0.034	0.201
GO:0002376	immune system process	1	1383	4	0.035	0.200
GO:0016044	cellular membrane organization	2, 4	320	2	0.037	0.202
GO:0061024	membrane organization	3	322	2	0.037	0.203
GO:0009790	embryo development	3	854	3	0.042	0.208

Analysis was performed with human homologs using the Manteia web tool.

Note: Each promoter can represent more than one gene.

* Only 6 GO terms were found
