# Supplementary material for: Large Sex Differences in Chicken Behavior and Brain Gene Expression Coincide with Few Differences in Promoter DNA-Methylation
Source: PLoS One. 2014 Apr 29;9(4):e96376. doi: 10.1371/journal.pone.0096376 (PMC4004567; doi:10.1371/journal.pone.0096376)
Supplement: Table S5 — Mean values of the behavior variables recorded in each of the behavioral test. (PDF) [file pone.0096376.s006.pdf]

**Table S5**. Mean values of the behaviour variables recorded in each of the behavioural tests.

| | Females | | | Males | | | p-value |
|---|---|---|---|---|---|---|---|
| | **Mean** | | **SEM** | **Mean** | | **SEM** | **Sex** |
| ***Social Reinstatement 3W (SR)*** | | | | | | | |
| duration social zone (s) | 107.8 | ± | 7.1 | 110.5 | ± | 6.8 | 0.693 |
| latency enter social zone (s) | 35.0 | ± | 4.5 | 29.0 | ± | 3.6 | 0.538 |
| mean distance moved (cm) | 1543.4 | ± | 52.3 | 1551.2 | ± | 46.9 | 0.862 |
| ***Open Field 4W (OF4)*** | | | | | | | |
| frequency center (per minute) | 3.8 | ± | 0.2 | 4.5 | ± | 0.2 | **0.030** |
| duration center (s) | 125.0 | ± | 4.3 | 135.9 | ± | 4.0 | 0.088 |
| latency center zone (s) | 55.7 | ± | 4.3 | 45.7 | ± | 3.7 | **0.024** |
| crossed zones (per minute) | 16.5 | ± | 0.8 | 16.5 | ± | 0.7 | 0.428 |
| distance moved (cm) | 2678.6 | ± | 106.7 | 2899.3 | ± | 98.8 | 0.066 |
| frequency corner (per minute) | 3.1 | ± | 0.2 | 3.0 | ± | 0.2 | 0.909 |
| duration corner (s) | 75.4 | ± | 3.4 | 65.8 | ± | 3.4 | **0.016** |
| frequency edges (per minute) | 6.9 | ± | 0.3 | 7.4 | ± | 0.3 | 0.133 |
| duration edges (s) | 97.9 | ± | 3.0 | 97.0 | ± | 2.8 | 0.804 |
| ***Foraging/Exploration test 13w (FE)*** | | | | | | | |
| Familiar food (% of picks) | 53.6 | ± | 2.2 | 74.8 | ± | 2.1 | **0.000** |
| Saw dust (% of picks) | 16.4 | ± | 1.0 | 11.2 | ± | 0.9 | **0.000** |
| Hidden food (% of picks) | 30.0 | ± | 1.9 | 15.7 | ± | 1.3 | **0.000** |
| ***Aeral predator 15w (AP)*** | | | | | | | |
| Explore (% of observations) | 17.1 | ± | 1.5 | 18.4 | ± | 1.7 | 0.895 |
| Groundpeck (% of observations) | 6.0 | ± | 0.9 | 7.2 | ± | 1.2 | 0.779 |
| Preening (% of observations) | 1.4 | ± | 0.3 | 2.8 | ± | 0.5 | **0.022** |
| Voc (% of observations) | 56.6 | ± | 2.8 | 44.2 | ± | 2.6 | **0.002** |
| Stand alert (% of observations) | 65.3 | ± | 2.6 | 66.3 | ± | 2.5 | 0.368 |
| Walk Alert (% of observations) | 58.2 | ± | 3.0 | 50.0 | ± | 3.0 | 0.177 |
| Freeze (% of observations) | 12.7 | ± | 2.4 | 11.6 | ± | 2.2 | 0.412 |
| Esc. Att. (% of observations) | 1.8 | ± | 0.3 | 0.7 | ± | 0.2 | **0.000** |
| ***Open field 16w (OF16)*** | | | | | | | |
| Crossed zones (per minute) | 2.7 | ± | 0.3 | 3.3 | ± | 0.3 | 0.062 |
| Frequency edges (per minute) | 1.1 | ± | 0.1 | 1.4 | ± | 0.1 | 0.095 |
| Frequency center (per minute) | 1.6 | ± | 0.2 | 1.9 | ± | 0.2 | 0.072 |
| ***Tonic Immobility 17w (TI)*** | | | | | | | |
| Ind attempts (nmb) | 2.4 | ± | 0.1 | 1.8 | ± | 0.1 | **0.001** |
| First movment (s) | 191.2 | ± | 21.5 | 128.5 | ± | 17.1 | 0.065 |
| Rigthening (s) | 358.0 | ± | 20.0 | 299.2 | ± | 19.5 | **0.015** |
| ***Undisturbed behaviour 27w (UB)*** | | | | | | | |
| Feeding (% of observations) | 10.8 | ± | 0.8 | 8.1 | ± | 0.8 | 0.099 |
| Explore (% of observations) | 35.4 | ± | 2.2 | 19.9 | ± | 1.8 | **0.000** |
| Active (% of observations) | 80.9 | ± | 3.9 | 42.5 | ± | 3.4 | **0.000** |
| Perch (% of observations) | 2.0 | ± | 0.6 | 16.2 | ± | 1.6 | **0.000** |
| Comfort (% of observations) | 10.6 | ± | 1.1 | 34.0 | ± | 2.4 | **0.000** |

| | | | | | | | |
|---|---|---|---|---|---|---|---|
| Jump/flight (% of observations) | 5.8 | ± | 0.9 | 9.0 | ± | 1.1 | **0.000** |
| Social pecks(% of observations) | 1.2 | ± | 0.2 | 0.7 | ± | 0.1 | 0.939 |
| Aggressive (% of observations) | 1.2 | ± | 0.1 | 1.2 | ± | 0.2 | 0.530 |
| observations) | 1.5 | ± | 0.1 | 1.1 | ± | 0.2 | 0.052 |
| Other Social (% of observations) | 0.5 | ± | 0.2 | 0.2 | ± | 0.0 | 0.938 |
