# Supplementary material for: Large Sex Differences in Chicken Behavior and Brain Gene Expression Coincide with Few Differences in Promoter DNA-Methylation
Source: PLoS One. 2014 Apr 29;9(4):e96376. doi: 10.1371/journal.pone.0096376 (PMC4004567; doi:10.1371/journal.pone.0096376)
Supplement: Table S6 — Primers used in qPCR and bisulfite sequencing. (PDF) [file pone.0096376.s007.pdf]

Table S6 - Primers used in qPCR and bisulfite sequencing

	Oligonucleotide sequence	Type	Target	5´ modification
Biseq				
	GGGTATTATTTAGTTTGTTGAGTGT	PCR-Forward	ZFR chromosome 1 promoter	Biotinylated
	AAAAACTATTCCCATAAATTCCAACAC	PCR-Reverse	ZFR chromosome 1 promoter	None
	CCCATAAATTCCAACACC	Sequencing	ZFR chromosome 1 promoter	None
	GTGGAAAGGTATAGATAATTAAGGGTAGT	PCR-Forward	MHM within predicted 2nd exon	Biotinylated
	CCCTTATTCTTACAATACTCTACCATCAC	PCR-Reverse	MHM within predicted 2nd exon	None
	CCTAAATATAATAAATACTTTTTCC	Sequencing	MHM within predicted 2nd exon	None
	ATGATGTTGATGTGGTTATAGTTG	PCR-Forward	MHM Upstream promoter within repeat	None
	TCCAATACTTCATCCATAACACTTT	PCR-Reverse	MHM Upstream promoter within repeat	Biotinylated
	GTTATTTTTTTTAGTTATTAGATGG	Sequencing	MHM Upstream promoter within repeat	None
qPCR				
	CTATCAACAGCCGACAGCAA	PCR-Forward	ZFR chromosome 1 exon 2-3	None
	GCTGGGCTTGGGTATATTGA	PCR-Reverse	ZFR chromosome 1 exon 2-3	None
	GGAGCCAAGCATCAGAAAGT	PCR-Forward	ZFR chromosome Z exon 7-8	None
	GTTTGCCGTGGGAGTAAGAA	PCR-Reverse	ZFR chromosome Z exon 7-8	None
	CGCTGCCATCTGGTAACTGG	PCR-Forward	MHM novel gene predicted exon 2-3	None
	CCTGGAAAGAAAAGCACTTGGA	PCR-Reverse	MHM novel gene predicted exon 2-3	None
	AACTCTCCGCCTGCCCCTTT	PCR-Forward	First EST downstream MHM novel gene	None
	CCAGAGGTGTGGGTCTGTGG	PCR-Reverse	First EST downstream MHM novel gene	None
	TGTCAAAACCAGCCGTTTCC	PCR-Forward	Intronic EST between exon 1 and 2 of MHM novel gene	None
	TTCCACCCTCTCTCCCCACT	PCR-Reverse	Intronic EST between exon 1 and 2 of MHM novel gene	None
